# Predictors of Children's Secondhand Smoke Exposure at Home: A Systematic Review and Narrative Synthesis of the Evidence

**DOI:** 10.1371/journal.pone.0112690

**Published:** 2014-11-14

**Authors:** Sophie Orton, Laura L. Jones, Sue Cooper, Sarah Lewis, Tim Coleman

**Affiliations:** 1 UK Centre for Tobacco & Alcohol Studies & Division of Primary Care, University of Nottingham, Nottingham, United Kingdom; 2 UK Centre for Tobacco & Alcohol Studies and Unit of Public Health, Epidemiology & Biostatistics, School of Health & Population Sciences, University of Birmingham, Birmingham, United Kingdom; 3 UK Centre for Tobacco & Alcohol Studies & Division of Epidemiology & Public Health, University of Nottingham, Nottingham, United Kingdom; Harvard Medical School, United States of America

## Abstract

**Background:**

Children's exposure to secondhand smoke (SHS) has been causally linked to a number of childhood morbidities and mortalities. Over 50% of UK children whose parents are smokers are regularly exposed to SHS at home. No previous review has identified the factors associated with children's SHS exposure in the home.

**Aim:**

To identify by systematic review, the factors which are associated with children's SHS exposure in the home, determined by parent or child reports and/or biochemically validated measures including cotinine, carbon monoxide or home air particulate matter.

**Methods:**

Electronic searches of MEDLINE, EMBASE, PsychINFO, CINAHL and Web of Knowledge to July 2014, and hand searches of reference lists from publications included in the review were conducted.

**Findings:**

Forty one studies were included in the review. Parental smoking, low socioeconomic status and being less educated were all frequently and consistently found to be independently associated with children's SHS exposure in the home. Children whose parents held more negative attitudes towards SHS were less likely to be exposed. Associations were strongest for parental cigarette smoking status; compared to children of non-smokers, those whose mothers or both parents smoked were between two and 13 times more likely to be exposed to SHS.

**Conclusion:**

Multiple factors are associated with child SHS exposure in the home; the best way to reduce child SHS exposure in the home is for smoking parents to quit. If parents are unable or unwilling to stop smoking, they should instigate smoke-free homes. Interventions targeted towards the socially disadvantaged parents aiming to change attitudes to smoking in the presence of children and providing practical support to help parents smoke outside the home may be beneficial.

## Background

Exposure to secondhand smoke (SHS), also known as passive smoking or environmental tobacco smoke, is the involuntary inhalation of other people's cigarette smoke. Children's exposure to SHS has been causally linked to increased risks of respiratory tract infections, middle ear infections, sudden unexplained death in infancy, and asthma [1]. A World Health Organisation (WHO) consultation report in 1999 concluded that SHS was a substantial threat to child health, with the 2006 US Surgeon General report later arguing that there is no safe level of SHS exposure [2].

Forty percent of children globally are exposed to SHS [3]. The two main determinants of children's SHS exposure in England have been reported to be smoking by parents or caregivers, and whether smoking occurs in the home [4], [5]. Smoke-free legislations banning smoking in enclosed public places have been widely introduced, with a reported 109 countries having implemented legislations by 2012 [6]. However such legislations do not cover smoking in private residences [1]. Children who spend a large proportion of their time indoors [7] and in close proximity to smoking parents [8], [9] are particularly at risk of SHS exposure in the home. In the UK, around two million children are estimated to be exposed to SHS in the home [1], with 52% of children who live with one or more smoking parents being regularly exposed [10]. Similar findings were reported in the 2006 Global Youth Tobacco Survey, where internationally 46.8% of never smoking young people aged 13–15 years were exposed to SHS in the home in the last seven days, with the highest level of exposure observed in Europe at 71.5% [11].

To our knowledge, studies which aim to understand the factors or characteristics associated with children's SHS in the home have not been previously reviewed. Consequently, we have carried out such a review of relevant studies conducted in children aged ≤18 years, examining factors associated with home SHS exposure. We aimed to identify factors, such as environmental or socioeconomic characteristics, which have been shown to be independently associated with children's SHS exposure in the home, and to determine potential characteristics that may be important for the development of effective future SHS and smoke-free home interventions.

## Methods

This systematic review was conducted and reported in accordance with the PRISMA guidelines [12].

### Systematic Review Methods

Electronic databases MEDLINE, EMBASE, PsychINFO, CINAHL and Web of Knowledge were searched to the end of July 2014 without date restrictions, using combinations of the following key words: secondhand smoke, environmental tobacco smoke, passive smoke/smoking, smoking in the home, smoke-free home, smoking rules, child, children, school child*, infant, baby, babies, parent, mother, father, predictor, association, factors, determinants.

The reference lists of papers identified as being relevant in the above electronic searches were also hand searched.

### Inclusion and exclusion criteria

Titles and abstracts identified from the searches were reviewed, and all studies meeting the following inclusion criteria identified: (a) English language studies examining the factors associated with SHS exposure in children aged ≤18 years, (b) reported a measure of child SHS exposure (e.g. parent reported exposure in the home; child self-reported exposure in the home; objective measures, biochemically validated exposure such as cotinine, carbon monoxide; home air particulate matter), (c) examined potential factors/associations for child SHS exposure (e.g. demographic, social/environmental, pregnancy factors, post-partum factors, health/emotional, tobacco related, smoking in pregnancy behaviours).

The age cut-off of ≤18 years for childhood was chosen to reflect variation in the legal age of adulthood across countries, with the majority of countries considering those aged 19 to be adults, and was considered appropriate as it is also the upper-limit at which adolescents are likely to remain in compulsory full-time education.

Whilst biomarkers are able to provide a quantitative measure of SHS exposure, this may reflect exposure both in the home and elsewhere. However, there is strong evidence to suggest that biomarkers can be used as an appropriate measure for child domestic SHS exposure. Research has shown that children spend the largest proportions of their time either in school attendance or as leisure time inside the home [13], with a reported 75–80% of their time spent in the home [14], [15]. This, coupled with the widespread implementation of smoking bans in enclosed public places, makes the home the primary source of SHS exposure [4], [5]. Furthermore, previous research has found biomarkers and reported child SHS exposure specifically in the home to have strong and consistent correlations across a range of ages (r range  = 0.36–0.66) [16]–[18]. Similarly, papers that used self-reported measures of indoor SHS exposure, for example, smoking in the same room as children, were included in this review on the assumption that most of this indoor exposure would occur in the home.

Papers that were not original quantitative methodologies were excluded. Papers exploring associations with parental reported ‘smoke-free homes’ (e.g. their child was NOT exposed to SHS in the home) were also excluded; creating ‘smoke-free homes’ is a behaviour change, and thus it is likely that there are a number of complex reasons, barriers or facilitators related to implementing home smoking bans. The factors associated with these are therefore likely to be quite different to those associated with children's SHS exposure in the home.

Following the title and abstract review, SO (first author) independently reviewed the full texts. A summary of each of the included studies is presented in Table S1. The significant associations (using the significance level adopted by each individual study) and adjusted sizes of effect of associations in each study were further compiled into a separate table (Table S2). In papers using numerous measures of SHS of exposure, the outcome that related specifically to SHS in the home was used where possible. The purpose of this review was to identify, rather than quantify, the factors and characteristics associated with children's SHS exposure in the home; a meta-analysis was therefore considered inappropriate and data were synthesised in a narrative review.

### Assessment of Methodological Quality

Studies that met the inclusion criteria were assessed for quality using a modified version of the Cochrane Collaboration Non-Randomized Studies Working Group recognised Newcastle-Ottawa Quality Assessment Scale [19], [20]. Herzog and colleagues [20] modified the original Newcastle-Ottawa Quality Assessment Scale for use when assessing the quality of cross-sectional studies. The studies in this review were all cross sectional in design and so using these criteria, studies were critically appraised and awarded a quality rating score out of a maximum of ten (Table S1). An *a priori* cut off point of seven points out of a possible 10 was used to identify papers considered to be of higher methodological quality, as has been used previously with the comparable original scale [21]–[23]. All studies of both low and high quality were included in the review, with study quality used to inform the results and conclusions made throughout.

## Results

**Figure 1:**
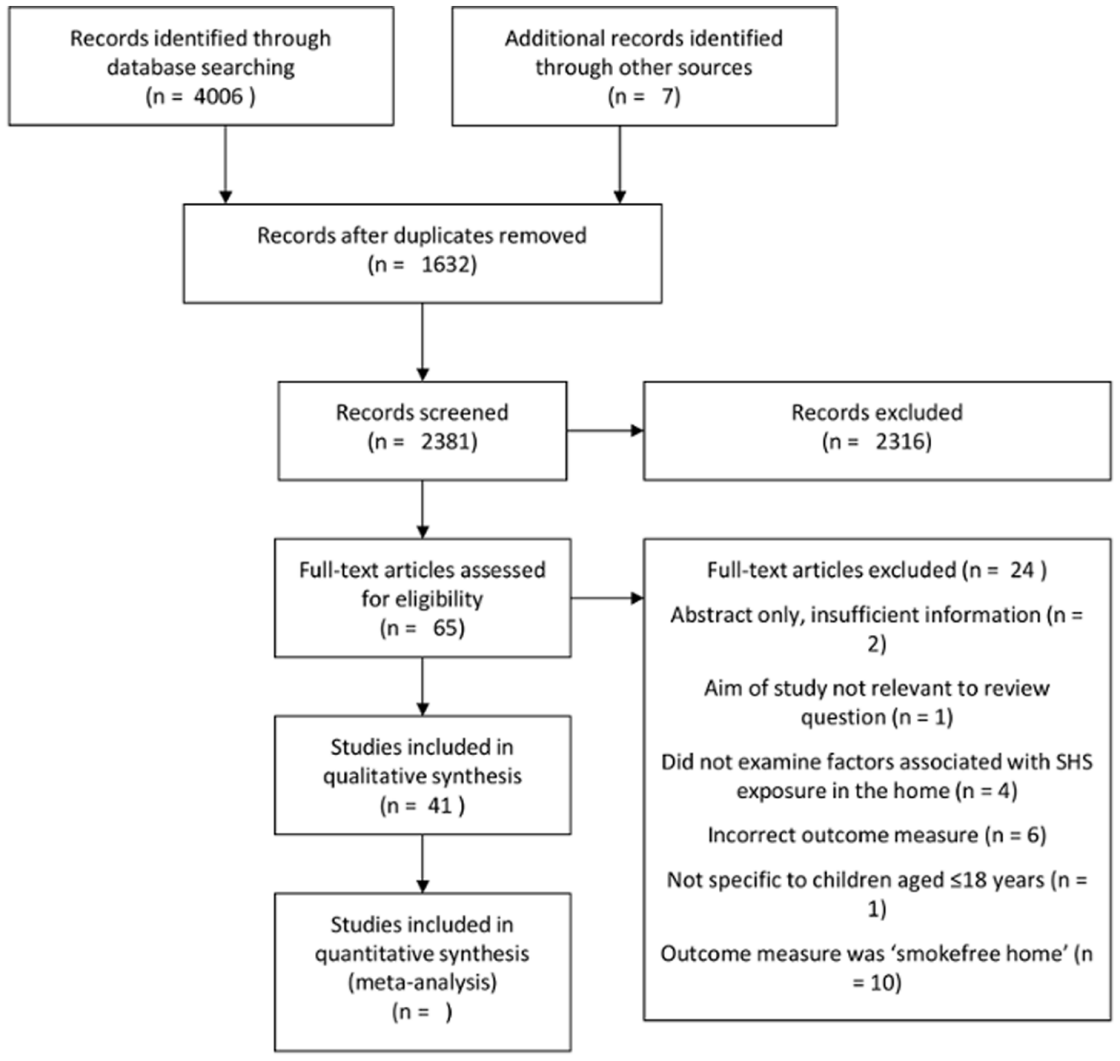
Systematic search results flow diagram of included and excluded studies.

There were 4013 papers identified through the systematic literature searches. After removal of duplicates, a further 2,316 articles were excluded based on title and abstract review. These included intervention studies to reduce child SHS exposure, studies examining the health risks associated with child SHS exposure and editorial papers. Sixty-five papers were considered as potentially eligible based on title and abstract review, and full-texts were obtained. Following the review of full-texts, 41 of these papers were included in the final review (Figure 1).

### Included studies

#### Location

Ten of the 41 studies were conducted in the UK (England [5], [24]–[26], Scotland [27]–[30], Wales [31], England and Wales [32]), eight in the USA [33]–[40], three in Germany [41]–[43], three in Greece [44]–[46], two in Korea [47], [48] and one each in Denmark [49], Sweden [50], Finland [51], Norway [52], Italy [53], Spain [54], Puerto Rica [55], Australia [56], Malaysia [57], Mongolia [58], South Africa [59], India [60], Taiwan [61], Thailand [62] and Tehran [63].

#### Study design

Thirty of the papers reported studies which were of cross sectional design [24], [25], [30], [32], [34]–[39], [41], [44], [45], [47]-[50], [52]-[57], [60]-[62], six were reports of repeated cross sectional designs [5], [27], [28], [31], [33], [51] and three studies were cross sectional using samples recruited as part of intervention studies [29], [43], [63].

#### Assessment of quality

Using the modified Newcastle-Ottawa Quality Assessment Scale [19], [20], the median quality score of studies included was seven points (range 2–9). Twenty two papers [5], [25]–[32], [39], [44]–[47], [50], [53], [54], [56], [57], [59], [62]–[64] were considered to be of higher quality (Table S1). The remaining studies were of lower quality, primarily due to lower representativeness of study samples, low study power or limited control of potentially confounding factors within analysis.

#### Ages of children included

The majority of studies focused on school-age children of approximately 5–18 years [5], [25]–[28], [30]–[33], [37], [39]–[42], [46], [47], [51], [53], [57]–[59], [61], or a broader age range to include both preschool and school-aged children (≤18 years) [24], [34], [36], [38], [44], [45], [48], [49], [56], [64]. Eight studies focused on SHS exposure in younger children; five [29], [43], [50], [52], [54] of these examined SHS exposure in preschool-children aged less than five years, and only three [35], [62], [63] focussed on SHS exposure specifically in infants under two years of age.

#### Measures of SHS exposure

Eighteen studies used the following validated measures of child SHS exposure: salivary cotinine [5], [25], [27]–[32], [57], urinary cotinine [26], [37], [42], [44], [47], [50], [53], [54], [63], [64], serum cotinine [39] or airborne particulate matter[PM_2.5_] [29]. Some of these studies also included self-report measures, such as parents'/carers' [5], [29], [42], [44], [47], [50] or children's [27], [31] reports of home SHS exposure, or parent [32], [37] or child [28] reported SHS exposure outside of the home.

A number of other studies used only self-report measures such as parental [24], [33], [34], [36], [38], [40], [41], [43], [45], [48], [49], [52], [56], [61], [62] or child (11–17 years of age) [46], [59], [60] reported exposure in the home. Two studies used parental/respondent [35] or child self-reported [51] smoking in the same room as children, and a further study [58] used child reported SHS exposure in the home and elsewhere. As can be seen in Table S1, different definitions of reported child SHS exposure were used across the studies. These included reported home smoking restrictions or location of smoking at home [24], [34], [36], [38], [41], [43], [49], [61], hours per day child exposed [35], [51], number of days per week child exposed [33], [59], [60], number of cigarettes child exposed to [48], exposure to cigarettes in given time periods (i.e. 12 months [40], [56]; seven days [58], [62]), smoking in the home in front of children [45], [46], [52], or any smoking in presence of children [47].

#### Factors associated with child SHS exposure

Of the 41 included studies, the associations between 90 different variables and child SHS exposure were identified; these were grouped into five conceptually similar categories: (1) socioeconomic status (SES) (including composite measures of SES, income, employment and health insurance type), (2) parental characteristics (education, age, race/ethnicity), (3) family and home characteristics (family size, family structure, home environment), (4) child characteristics (age, gender), and (5) parental smoking characteristics (smoking behaviour, attitudes and self-efficacy). The size of effect of statistically significant associations reported between principle variables and SHS exposure in the home (using significance level reported by individual studies) are presented in Table S2.

#### (1) Socioeconomic status

The relationship between child SHS exposure and proxy measures of SES were examined in 11 studies; measures of SES used were the Registrar General's Social Class system [5], [28], [30], [32], [63], [65], area level deprivation indicators [47], [56], the Family Affluence Scale [27], [28], [31], [66], the Townsend score [25], [67] and wealth [62]. In ten out of 11 studies [5], [25], [27], [28], [30]–[32], [47], [56], [63] there were significant associations between low SES and increased exposure. This was observed both in studies using biomarkers as an outcome measure [5], [25], [27], [28], [30]–[32], [47], [63], and reported exposure [56]. Children of parents in lower SES groups were up to three times more likely to be exposed to SHS, with the odds ratios (OR) from individual studies ranging from 1.1 to 3.3. The majority of studies reporting this were of higher methodological quality [5], [25], [27], [28], [30]–[32], [47], [63].

Seven studies with mixed findings [36], [37], [40], [41], [47], [57], [61] investigated whether or not there was an independent relationship between income and child SHS exposure. Overall a significant association was reported in three studies [37], [41], [47]. Two studies [37], [57] used biomarkers as outcome measures, with just one [37] reporting a significant association. Five studies [36], [40], [41], [47], [61] relying on reported exposure as an outcome measure examined income, with two [41], [47] reporting a significant association between low income and child SHS exposure in the home. This finding did not differ according to study quality.

There was similarly inconsistent evidence for a link between employment status or occupation and child SHS exposure. Three studies found a significant association between employment and exposure to SHS in the home; in one study [5] using biomarkers as an outcome measure, children whose parents' employment status was ‘other’ (including looking after the home) had significantly higher salivary cotinine levels, however, those with unemployed parents did not. A second study [41] that used reported exposure as an outcome measure found a significant association between parental unemployment or part-time employment and increased child exposure. A third study [43], also using reported exposure as an outcome measure found children of households where only one parent was employed were at an increased risk. No significant association was observed in four studies [24], [34], [62], [64]. These findings did not vary dependent upon study quality. There was also little indication of a relationship between type of occupation and child SHS exposure, with just one study [57] reporting that children whose fathers were in the armed forces had higher levels of salivary cotinine compared to children whose fathers were in managerial or professional roles.

#### (2) Parental characteristics

Twenty-six studies [5], [24], [33]–[35], [37]–[45], [47]–[49], [51]–[54], [57], [61]–[64] investigated the relationships between parental or highest level of education within the household and child SHS exposure at home, with 18 [5], [24], [33], [35], [37]–[41], [43], [44], [47], [49], [52]-[54], [57], [62] reporting a significant association between low education and increased risk of exposure. In one study [54] there was a significant association between paternal education and child exposure, but no significant association with maternal education. Although there was variation in how parental education was measured and categorised, children whose parents had the lowest levels of education were up to ten times (OR range 1.08 to 10.4) more likely to be exposed to SHS. These findings did not differ according to study outcome measure or quality; of those reporting a significant association between parental education and child SHS exposure in the home, seven [5], [37], [39], [44], [53], [54], [57] used biomarkers as an outcome measure compared to 11 studies [24], [33], [35], [38], [40], [41], [43], [47], [49], [52], [62] using reported exposure. Of the high quality studies, three [45], [63], [64] found no significant association of education on exposure, whilst eight found a significant association [5], [39], [44], [47], [53], [54], [57], [62].

Parental race or ethnicity was examined in nine studies [5], [26], [33], [35], [36], [38], [39], [42], [50], with a significant association found in eight [5], [26], [33], [35], [36], [38], [39], [42] of these. In the UK, children of White parents had significantly higher SHS exposure, as measured by biomarkers, than children from other ethnicities [5], [26]. The association between race or ethnicity in USA based studies was less clear; there was some evidence that children of White parents were at an increased risk of SHS exposure [33], [35], [38]; however, other studies found significant associations between SHS exposure and other races/ethnicities [33], [36], [38], [39]. A German based study found children of non-German nationality to have significantly higher urinary cotinine levels [42]. One study [62] further found children of Muslim fathers to be significantly more likely to be exposed to SHS in the home. The outcome measure used across studies did not influence whether a significant association was observed, with four studies [5], [26], [39], [42] that used biomarkers as an outcome, and four [33], [35], [36], [38] that used reported exposure finding a significant association. However, five [33], [35], [36], [38], [42] of the studies reporting a significant association between ethnicity and child SHS exposure in the home were of lower quality.

Parental age was not shown to be linked to child SHS exposure; eleven studies [24], [29], [34]–[36], [45], [49], [61]–[64] explored this relationship, however only two [29], [35] found significant associations between lower parental age and measures of SHS exposure, and one [62] found a significant association with but with no clear direction of effect. This finding did not differ according to study outcome measure or quality.

#### (3) Parental smoking behaviour and attitudes

Of the 18 studies [5], [25], [27], [29], [30], [34], [36], [42], [43], [45], [46], [48], [51], [53], [54], [57]–[59] that investigated parental or household member cigarette smoking status, 15 [5], [25], [27], [30], [34], [42], [43], [45], [46], [48], [51], [53], [57]–[59] identified a significant association between this and SHS. Children of smoking mothers were up to seven times (OR range 2.1 to 6.9) more likely to be exposed in the home, and children of parents who both smoked were up to 13.5 times (OR range 2.9 to 13.5) more likely to be exposed in the home. This was observed both in studies using biomarkers as an outcome measure [5], [25], [27], [30], [42], [53], [57], and reported exposure [34], [43], [45], [46], [48], [51], [58], [59]. These findings did not differ according to study quality.

Eight studies examined an association between the number of cigarettes smoked by parents either per day [44], [45], [49], [50], [53], [61], [63] or per week [34] and child exposure. In four of these [44], [45], [50], [53] a significant association was observed; children whose parents had a higher level of cigarette consumption were more likely to be exposed to SHS. One study [45] observed a significant association with increased number of cigarettes smoked per day by the mother, but not the father. Two further studies [49], [61] looked at the effect of respondents being a daily smoker, however no significant association was reported. Significant associations between parental cigarette consumption and child SHS exposure was more frequently observed in studies using objective outcome measures [44], [50], [53] and in studies of high quality [44], [45], [50], [53].

The number of cigarettes smoked in the home was explored in a further four studies [29], [39], [42], [54], all of which used objective measures of SHS exposure. In three of these [29], [39], [42] there was a significant relationship between more cigarettes smoked in the home and child exposure; however, this was only investigated in a univariate analysis which means that this finding may not be independent of other confounding factors.

Four studies [29], [33], [52], [61] measured and reported significant associations between parental attitudes towards smoking and SHS exposure. These studies used reported exposure [33], [52], [61] and home airborne particulate matter [PM_2.5_] [29] as outcome measures. Although the measurement of attitudes varied across the studies, generally more negative attitudes towards SHS exposure were related to lower exposure. In three studies [33], [52], [61] there was an association between negative opinions towards SHS and reduced risk of exposure. In one study [33], agreement that SHS was harmful to health was associated with reduced risk of child SHS exposure in the home. One study [52] developed a scale of six questions measuring attitudes towards statements about the rights of adults to smoke in their own homes, the rights of children to live in smoke-free homes and the safety of SHS exposure; those with lower scores (reflecting negative attitudes towards child SHS exposure) were less likely to smoke in the home [52]. One study [61] found that those who agreed more with their family's anti-smoking reactions to smoking in the home were less likely to expose their children to SHS. A further study [29] observed lower maximum indoor particulate matter (PM_2.5_) concentrations and child salivary cotinine among those mothers who strongly agreed that they would ask a smoker to smoke outside their house; however, this was only found in univariate analysis and there was no significant effect for other attitudinal questions. Three of the studies [33], [52], [61] reporting a significant association between parental attitudes and child SHS exposure in the home were of lower quality. Two further studies [59], [60] found child attitudes towards the harmfulness of SHS was associated with exposure in the home, however the direction of this association was unclear.

#### (4) Family and home characteristics

Thirteen studies [30], [34]–[38], [40], [41], [47], [48], [51], [61], [64] looked at a link between marital status or family structure and child SHS exposure. In five studies [30], [37], [38], [41], [64] being a single parent was associated with children's SHS. Further associations were found for exposure among children whose mothers were unmarried [35], who were separated [51] or part of a step-family [38], with children from these families being up to twice as likely (OR range 1.1 to 2.1) to be exposed to SHS. These findings did not differ between outcome measures used; significant associations between marital status and family structure were observed both in studies using biomarkers as an outcome measure [30], [37], [64] and reported child SHS exposure in the home [35], [38], [41], [51] However, five of the studies [35], [37], [38], [41], [51] reporting an association were of lower quality.

There was no clear relationship between family size and exposure, which was investigated in 11 studies [30], [35], [39]–[41], [43], [45], [47], [56], [62], [63]. In studies using biomarkers as an outcome measure, three [39], [47], [63] found no association whilst one study [30] reported child SHS exposure to decrease with increasing number of children in the family. There were mixed findings in studies using reported exposure; in three studies child SHS exposure in the home [35], [43], [56] was associated with 20–72% (OR range 1.2 to 1.72) increased odds of SHS with one or more siblings, or a larger family size, whilst in one study exposure decreased with increasing number of children in the family [41]. A further three studies found no significant association [40], [45], [62]. Those reporting a significant association tended to be of lower quality [35], [41], [43], [56].

There was some evidence for an association with accommodation size or characteristics. Seven studies [5], [30], [42], [43], [53], [54], [63] looked at crowding, defined as number of people per bedroom; four studies [5], [30], [42], [53] all using biomarkers as outcome measures found a significant relationship between more crowded homes and increased SHS exposure. The only study [43] to use reported exposure as an outcome measure found no significant association between child SHS exposure in the home and crowding, however this study was also of lower quality. There was no evidence that this was influenced by study quality. There was similarly some evidence for a relationship between size of home and exposure, which was only measured in studies using biomarkers as outcome measures. Increased home floor surface area was significantly associated with lower SHS exposure in two studies [42], [44], and fewer rooms being associated with an increased risk of exposure in a third study [39]. No association with accommodation size was found in a further study [50]. Other significant relationships included the use of air conditioning in the home [57] and the availability of outside space [36], [43] both being associated with reduced child exposure. These findings did not differ according to study quality.

#### (5) Child characteristics

The association between child age and exposure was explored in 19 studies [5], [24], [25], [29], [32], [36], [38], [39], [44]–[46], [48], [53], [54], [58]-[60], [63], [64]. Nine of these [5], [25], [29], [32], [36], [39], [44], [45], [64] found younger children to be significantly more likely to be exposed to SHS in the home, or to have higher exposure. The studies reporting this association tended to use objective outcome measures [5], [25], [29], [32], [39], [44], [64], and to be of higher quality [5], [25], [29], [32], [39], [44], [45], [64] than those finding no significant association. Three studies [46], [58], [63] found the opposite association; one study [63] found urinary cotinine to increase significantly per one month increase in age among infants aged under one year, and two studies [46], [58] found older teenagers to be more likely to report SHS exposure in the home than younger teenagers. These findings did not differ according to study quality.

Nineteen studies [5], [29], [30], [32], [38], [39], [42], [44]–[47], [53], [54], [57]–[59], [62]–[64] looked at child gender and SHS exposure, with limited support for an association. Significantly higher salivary [5], [30], [32] and urinary cotinine [44] in females was observed in four studies. A further study [46] found female adolescents to be more likely to report smoking in their homes, however the remaining studies [29], [38], [39], [42], [45], [47], [53], [54], [57]–[59], [62]–[64] found no significant association. These findings did not differ according to study quality.

## Discussion

Children whose parents are smokers, are of low SES or less educated were at an increased risk of SHS exposure in the home. There was also some evidence that children whose parents held more negative attitudes towards SHS were less likely to be exposed. Associations were strongest for parental cigarette smoking status; compared to children of non-smokers, those whose mothers or both parents smoked were between two and 13 times more likely to be exposed to SHS at home. These findings show that the best way to prevent child SHS exposure in the home is by encouraging smoking parents to quit.

Literature in this review was synthesised narratively, which may introduce some bias if findings of one study are given inappropriate weight compared to others [68]. However, efforts were made to avoid such biases through methodically identifying papers, data extraction, and quality assessments of studies informing the synthesis of findings. It is further acknowledged that only one author reviewed and extracted data from papers. Previous research has reported single-reviewer data extraction to be at greater risk of error compared to multi-reviewer extraction [69]. However, this was found using reviewers who were unfamiliar with the topic area, and errors identified were found to be minimal and to have no significant impact on findings [69]. Papers using biomarkers as an outcome measure were included in this review; biomarkers are not able to identify the location in which exposure occurs, and it is therefore not possible to rule out that some exposure in these studies occurred in locations outside of the index home, such as in other people's homes and private vehicles. However, there is evidence of strong correlations between biomarkers and reported SHS exposure in the home [16]–[18], [70], so it is likely that associations between characteristics identified in this review and biomarkers are principally determined by home exposure.

There were a number of limitations inherent in the studies included in the review. Using a modified Newcastle-Ottawa Quality Assessment Scale [19], [20], 19 studies were considered of lower quality, primarily due to low representativeness of study samples and limited control of potentially confounding factors within analysis. Some studies were also at risk of low power and chance findings, whereby the authors used small sample sizes and examined multiple risk factors within their analyses. Furthermore, the studies included in this review were carried out in a broad range of different countries, and so there are likely to be wide cultural differences. These limitations may explain disparities in associations observed across studies, and should be taken into consideration when interpreting the findings of this review.

The finding that parental and other household member cigarette smoking status was associated with child SHS exposure supports previous research, which has shown the primary source of child SHS exposure to be smoking by parents [4], [71]. The greatest observed risks in this review were for children whose mothers [5], [25], [34], [51], [58] or both parents [5], [51], [58] were smokers, which strongly suggests that the best way to reduce child SHS exposure in the home is for parents who smoke to quit. This finding has implications for younger children of pre-school age, who spend an increased proportion of their time at home with parents compared to older, school-aged children [8], [9]. There was some evidence in this review that younger children may be at an increased risk of SHS exposure in the home, which was found in some high quality papers using biomarkers as outcome measures of SHS exposure. Research has found no significant differences in the elimination half-life of urinary cotinine between younger and older children, suggesting that higher cotinine levels observed in younger children are likely to be due to increased exposure [72].

In line with the findings of this review, sociodemographic characteristics are often linked to health inequalities. Low SES is frequently reported to be associated with poorer health outcomes, health morbidity and mortality [73]. Those with lower education have similarly been found to engage in fewer health promoting behaviours [73], [74], and have a higher smoking prevalence than more educated populations [75], [76]. There was some evidence in this review that children whose parents were single, separated or divorced were at an increased risk of SHS exposure in the home; children from single parent families [77]–[79], or whose parents/carers are unmarried [80] have also been shown to have worse health outcomes compared to those from traditional nuclear families. Previous research has shown single mothers to be more likely to relapse to smoking after pregnancy [81], and unmarried or divorced adults to be more likely to be daily smokers [82] or heavier smokers [83].

In a recent review [84], the effectiveness of any one interventional approach to reduce children's SHS exposure was not conclusively demonstrated and as such there remains a need for novel, evidence-based interventions which are sensitive to both the context in which smokers live and smokers' environments. Whilst the demographic characteristics found to be associated with children's SHS exposure in the home are not easily modifiable [85], they may help to inform which children, parents or families are best targeted in future interventions. For example, this review suggests that interventions targeted towards low SES groups aiming to promote smoking cessation would have a positive impact on children's exposure in the home. Where parents are unable or unwilling to quit smoking, making the home smoke-free is the only effective way to protect children from SHS exposure [50], [86]–[88]. The Theory of Reasoned Action argues that interventions designed to change beliefs and attitudes can influence intentions and subsequent behaviour across a range of health behaviours [89]. Interventions targeting attitudes towards SHS by supporting parents to recognise the benefits of protecting their children from SHS may therefore be useful to promote smoke-free homes. However, previous research has shown home smoking behaviours to be complex and fluid among a group of disadvantaged parents [90]; changing attitudes alone may not be sufficient to change behaviour. A combined approach that targets attitudinal change and provides practical context specific advice to parents, for example balancing child safeguarding with smoking outside of the home or negotiation with other household smokers, may be helpful.

Future research is needed to explore SHS exposure specifically in very young infants; just three studies in this review explored factors associated with SHS exposure in this age group. Although other studies included infants of less than two years of age within their samples, this younger age group was not considered or reported independently of older children. Very young infants under two years of age may be particularly susceptible to the risks of SHS exposure as they have a higher respiration rate [91], [92], and underdeveloped lungs [93], [94]. This increased susceptibility can have serious health implications; infants exposed to SHS postnatally are more vulnerable to infections requiring hospitalization [95], have poorer respiratory health, including episodes of wheeze [96], [97], lower respiratory infection [96] and chronic bronchitis [97], and are at an increased risk of sudden unexpected death in early infancy [1]. The findings of this review suggest that younger infants could be at an increased risk of SHS exposure, though it is not possible to generalise other observed associations to very young infants based on the currently available literature.

## Conclusions

Children whose parents are smokers, are of low SES, less educated, or hold less negative attitudes towards SHS are at an increased risk of SHS exposure in the home. The largest observed risks were for children living in households with smokers; the best way to reduce child SHS exposure in the home therefore is for smoking parents to quit. If parents are unable or unwilling to stop smoking, they should instigate smoke-free homes. Interventions targeted towards socially disadvantaged parents aiming to change attitudes to smoking in the presence of children, and providing context specific practical support to help parents overcome barriers to smoking outside the home may reduce children's domestic SHS exposure.

## Supporting Information

Table S1 **Study characteristics.**
(DOCX)

Table S2 **Associations identified and strength of effect.**
(DOCX)

Checklist S1 **PRISMA 2009 Checklist.**
(DOC)
