# Supplementary material for: Predictors of Children's Secondhand Smoke Exposure at Home: A Systematic Review and Narrative Synthesis of the Evidence
Source: PLoS One. 2014 Nov 14;9(11):e112690. doi: 10.1371/journal.pone.0112690 (PMC4232519; doi:10.1371/journal.pone.0112690)
Supplement: Table S1 — Study characteristics. (DOCX) [file pone.0112690.s001.docx]

| **Author, year, location,**  **Newcastle-Ottawa quality rating^** | **Design, Data collection years** | **Recruitment, response rate** | **Sample size, age range of children** | **Main outcome measure (for purposes of review)** | **Associated factors examined in analysis**  ***confounders controlled for** | **Analysis **** | **Significant associations identified in multivariate analyses**  **(† univariate analysis only)** |
| --- | --- | --- | --- | --- | --- | --- | --- |
| Abidin et al. 2011[[57](#_ENREF_57)]  Malaysia  Quality rating:  8 | Cross sectional survey  April – September 2009 | Children from a minimum of 2 classrooms from years 4 and 5 within 24 National Schools across Kuala Lumpur and 3 rural districts in Negeri Sembilan invited to participate.  Saliva samples collected from 38.3% of invited participants | N = 1064  10-11 years of age | Salivary cotinine | Location (rural/urban)*  Parental cigarette smoking status*  Parental reported exposure*  Child gender  Paternal education (diploma/technical certificate, degree/college)*  Paternal occupation (armed forces, manager/professional)*  Family income (low, middle, high)  SHS in household (non-smoking, smoking)  Child’s sleeping area (own room/share with siblings, living room, share with parents/adults)  Use of air conditioner*  Use of exhaust system  Smoking restriction in home (total, partial, none) | Log salivary cotinine used.  Chi square tests  Multiple linear regression | Location (rural/urban)  Parental cigarette smoking status  Paternal occupation  Paternal education  Parental reported exposure  Use of air conditioner in home |
| Akhtar et al. 2010[[28](#_ENREF_28)]  Scotland  Quality rating:  9 | Repeated cross sectional survey  Jan 2006 – Jan 2007 | CHETS study (Changes in Child Exposure to Environmental Tobacco Smoke)^[^[^98^](#_ENREF_98)^]^,  Two nationally representative primary school classes in the same schools pre and post smoke-free legislation.  2006: 86% response rate. > 95% valid cotinine sample.  2007: 85% response rate. > 95% valid cotinine sample. | Questionnaire:  2006 N = 2532  2007 N = 2389  Saliva samples available for:  2006 N = 2403  2007 N = 2270  Approximately 11 years of age | Salivary cotinine.  Child reported parental cigarette smoking status: ‘do any of the following people smoke? Father, mother, stepfather, stepmother. Parental figures classes as smokers when described as smoking ‘every day’ or ‘sometimes’. Children then classified as living with ‘none’, ‘one (father figure only), one (mother figure only) or ‘two’ smokers. | Family socioeconomic classification (parental occupation coded into 1: I professional occupations & II managerial & technical. 2: IIIN skilled non-manual and IIIM skilled manual. 3: IV partly skilled and V unskilled. 4: economically inactive)  Family affluence scale (FAS)  Analysis controlled for:  Child age  Number of parents who smoke | Log salivary cotinine used.  Chi square tests  ANOVA  Linear regression | Socioeconomic status  Family affluence  Year (pre/post legislation) |
| Akhtar et al., 2009[[27](#_ENREF_27)]  Scotland  Quality rating:  9 | Repeated cross sectional survey  Jan 2006 – Jan 2007 | Recruited through the CHETS study (changes in child exposure to environmental tobacco smoke),[[98](#_ENREF_98)]  Two nationally representative primary school classes.  2006: 86% response rate, > 95% provided valid cotinine sample.  2007: 85% response rate, > 95% Provided valid cotinine sample. | Questionnaires:  2006 N = 2559 (86%)  2007 N = 2424 (85%)  After exclusion for missing data, final questionnaire data sets were:  2006 N = 2532  2007 N = 2389  Saliva samples available for:  2006 N = 2403  2007 N = 2270  Approximately 11 years of age | Salivary cotinine.  Child reported parental cigarette smoking status: ‘do any of the following people smoke? Father, mother, stepfather, stepmother. Parental figures classes as smokers when described as smoking ‘every day’ or ‘sometimes’. Children then classified as living with ‘none’, ‘one (father figure only), one (mother figure only) or ‘two’ smokers.  Child reported smoking restrictions in the home. ‘Is smoking allowed inside your home’ (categorised as complete restrictions, partial restrictions or no restrictions). | Number of parents smoking  Family affluence (Family Affluence Scale)  Analysis controlled for:  Age  Family SES | Log salivary cotinine used.  Chi square tests  Multinomial logistic regression  Linear regression analysis | Parental smoking  Time (pre/post legislation)  Child reported type of home smoking restrictions  Family affluence  Home smoking restriction type and survey year interaction  Home smoking restriction type and presence of parental smokers interaction |
| Alwan et al. 2010[[24](#_ENREF_24)]  England  Quality rating:  6 | Cross sectional survey  June 2008 | Sampled natural community neighbourhoods within Leeds, England. Sampled consecutive houses within these areas until over 310 households with children aged 0-16 years had completed the survey.  Response rate 50.9%. | 318 households  < 16 years of age | Home smoking restrictions: ‘if there are smoker(s) in your household, where does smoking take place?’ 1) in the presence of children, 2) any part of the house, 3) in the house but only if windows are open, 4) inside the house but only in a specific room, 5) only in a specific rooms with the windows open in that room, 6) only outside the house. | Head of household characteristics:  Age*  Male gender  Employment status (unemployed)*  Education (qualification)* | Chi-squared test  Multiple Logistic regression | Unemployed  Education (qualification below A-level) |
| Anuntaseree et al. 2008[[62](#_ENREF_62)]  Thailand  Quality rating:  7 | Cross sectional  October 2001 – August 2003 | Data collected as part of the Prospective Cohort Study of Thai Children. Cohort of infants born over a 1 year period in each of the 5 regions of Thailand recruited.  Response rate 76.7% | N = 3256  1 year of age | Respondent reported: ‘in the preceding week, did anyone in the household smoke in the same room as the infant?’ If yes, ‘did the father smoke in the same room as the infant, did the mother smoking in the same room as the infant, did any other family member smoke in the same room as the infant?’ | Paternal age*  Paternal education (primary school, secondary school, college or university)*  Religion (Buddhist, Muslim, Christian, Other)*  Occupation (professional, non-professional, unemployed)*  Economic status (poor, sufficient, wealthy)*  Child gender  Birth weight  Parity* | Chi-squared test  Multiple logistic regression | Paternal age  Paternal education  Religion |
| Baheiraei et al. 2010[[63](#_ENREF_63)]  Tehran  Quality rating:  7 | Cross sectional. Data from RCT.  2008 | Smoking households attending a health centre in southern Tehran  Response rate not reported | N = 130  < 1 year of age | Urinary cotinine (≥30 ng/ml indicating SHS exposure) | Infant age*  Infant gender  Infant weight  Breastfeeding  Maternal age  Paternal age  Maternal education (none/elementary, middle/high school, diploma or higher)  Paternal education (none/elementary, middle/high school, diploma or higher)  Maternal occupation (housewife, employed)  Social status (employer and junior employees or lower, skilled workers, semiskilled or unskilled worker*  Type of housing (homeowner, rent, other)  Car ownership  Number of children  Crowding index  Access to outdoor area  Separate room for infant  Daily number of cigarettes smoked  Parental report of infant SHS exposure  Day of urine collection  Nicotine dependence (Fagerstrom test)  Smoking restrictions at home* | Multiple logistic regression | Infant age  Social status |
| Bakoula et al. 1997[[44](#_ENREF_44)]  Greece  Quality rating:  8 | Cross sectional  Nov 1991 – April 1992 | Over a 6 month period, every fourth child that contacted the out-patient clinic of the Children’s University Hospital enrolled.  Response rate 99.7%.  Invalid urinary cotinine samples N = 4. | N = 2108 children   ≤14 years of age | Urinary cotinine  Parental reported number of cigarettes smoked in an average day while the child is at home, by either or both parents. | Child age*  Child gender*  Day of week cotinine sampled*  Floor surface area*  Central heating*  Maternal education (years)*  Paternal education (years)*  Parental smoking per day*  Precautions taken* | Log urinary cotinine used  Multiple linear regression | Child age  Gender  Day of the week  Floor surface area  Central heating  Maternal education  Paternal education  Parental smoking (cigarettes per day)  No smoking precautions |
| Bleakley et al. 2014[[36](#_ENREF_36)]  USA  Quality rating:  6 | Cross sectional survey  May – June 2012 | Random digit dialling of households in low-income areas in Philadelphia USA, identified to have child under the age of 13.  Response rate 25.3% | N = 456  <13 years of age | Parental reported child SHS exposure in the home: full smoking ban, no smoking in the presence of children, no ban/smoking allowed in the presence of children. | Race*  Income*  Gender*  Age*  Marital status (married/other)*  Child under 5 years*  Child ever diagnosed with asthma*  Number of smokers in household*  Who smokes in household (mother, father, other)*  Number of rooms*  Outdoor space available*  Who in the home is a smoker (father, mother, both parents, other)*  Exposure to anti-smoking advertisements*  Knowledge about the effects of SHS*  Smoking norms (friends of respondents who are smokers)* | Multinomial logistic regression | No home smoking ban, but smoking in the presence of children restricted:  Race  Child under 5 years  Child ever diagnosed with asthma  No home smoking ban, smoking allowed in the presence of children:  Race  Child under 5 years  Outdoor space available |
| Bolte & Fromme, 2009[[41](#_ENREF_41)]  Germany  Quality rating:  5 | Cross sectional survey  Wave 1: 2004-2005  Wave 2: 2005-2006 | Data collected during compulsory school entrance health examinations in three rural and three urban regions of Germany.  Response rate wave 1: 78%  Response rate wave 2: 73% | N = 12422 children  5-7 years of age | Parental reported child exposure at home, in cars and at hospitality venues.  ‘Is there smoking in the flat where your child lives?’  1)yes, inside the flat, 2) yes, but exclusively on the balcony or terrace, 3) no  Average number of cigarettes smoked daily by mother, father and other persons in the flat (including balcony or terrace) | Family size*  Single-parent family*  Nationality of child*  Parental education (very high, high, middle, low)*  Parental employment status*  Household equivalent income*  Study region* | Multiple Logistic regression | Family size  Single parent family  Nationality of child  Parental education  Parental employment status  Household equivalent income  Study region |
| Chen et al.2011[[37](#_ENREF_37)]  USA  Quality rating:  6 | Cross sectional  October 2006 – March 2008 | Recruited from outpatient clinics in Michigan, USA. For mothers with more than one child, child selected for participation on mother’s preference.  Response rate = 80% | N = 397  6-10 years of age | Urinary cotinine(≥10 ng/ml indicating SHS exposure)  Maternal reported child SHS exposure and duration in enclosed spaces | Marital status (single, not single)  Maternal education (≤high school, > high school)  Household income (≤ $2500, ≥$2501)  Number of prenatal check-ups (≤12, 13-14, ≥15)  Parental satisfaction (satisfied, not satisfied)  Controlled for maternal age, age of children, maternal race, child’s birth order | T tests  ANOVA  Chi square tests  Multiple logistic regression | Urinary cotinine:  Marital status  Maternal education (≤high school, > high school)  Household income (≤ $2500, ≥$2501)  Parental satisfaction |
| Cook et al.[[32](#_ENREF_32)]  England and Wales  Quality rating:  8 | Cross sectional survey  January – July 1990 | 10 towns in England and Wales selected (5 with high adult cardiovascular mortality, 5 with low adult cardiovascular mortality). 10 schools in each town recruited from.  Response rate with complete data 52.2% | N = 2721  5-8 years of age | Salivary cotinine  Parental reported current smoking habits | Child gender  Child age  Day of week saliva sample taken  Social class (Registrar General’s classification)  Town  Adjusted for mother’s smoking habits, father’s smoking habits, smoking by other household members | Geometric mean salivary cotinine used  Cross tabulations  Multiple linear regression | Child gender  Child age  Day of week saliva sample taken  Social class |
| Dell’Orco et al. 1995[[53](#_ENREF_53)]  Italy  Quality rating:  8 | Cross sectional  1990 – 1991 | Children attending 5^th^ grade in 7 randomly selected primary schools and all children attending secondary schools in the Latium region invited to participate. | N = 1199  12-15 years of age | Urinary cotinine | Child gender  Child age  Paternal education (years)*  Paternal occupation (non-manual, manual, not employed)  House size (rooms)  Household crowding (inhabitants per room)*  Parental smoking (maternal and paternal cigarettes/day)*  Other smokers in home*  Day of examination*  Hours of exposure to smoking outside home in preceding days* | Geometric mean urinary cotinine used  ANOVA  Multiple linear regression | Current parental smoking  Other smokers in household  Household crowding  paternal education  Day of examination  Hours of exposure outside home |
| Delpisheh et al. 2006[[25](#_ENREF_25)]  England  Quality rating:  8 | Cross sectional  1993-2001 | Systematic recruitment of children on class registers at 10 primary schools in low socio-economic areas of Merseyside. | N = 245  5-11 years of age | Salivary cotinine (≥1 ng/ml indicating SHS exposure) | Maternal cigarette smoking*  Presence of a smoker in the household*  Child age (<7 years)*  Deprivation (Townsend score > +6)* | Chi square test  Analysis of variance  Backward stepwise logistic regression | Maternal cigarette smoking  Presence of a smoker in the household  Child age (<7 years)  Deprivation (Townsend score > +6) |
| Gonzales et al., 2006[[34](#_ENREF_34)]  USA  Quality rating:  5 | Cross sectional survey  Nov 2003 – April 2004 | Recruited from waiting rooms of a paediatric emergency room/urgent care clinic, a family practice and paediatric health care facility, and a special supplemental nutrition program for women infants and children clinic.  Overall response rate 75.4%. | N = 269 mothers.  2-12 years of age | Parental reported home smoking restrictions:  ‘Would you say family members and visitors can: a) smoke wherever they want in your home, b) smoke in certain rooms only, c) not smoke anywhere inside your home. | Maternal’s country of birth*  Maternal current cigarette smoking status*  Marital status*  Maternal age  Education (qualification)  Employment  % of US federal poverty threshold  Current cigarette smoking status  Cigarettes smoked per week  Proportion of friends who smoke  Other adult smoker in home | Chi squared test  Multiple logistic regression models using non-automated stepwise modelling techniques  Significance level p < 0.05, however variables reaching p ≤ 0.25 in univariate analysis were also included in multivariate analysis. | Mother’s country of birth (Mexico/USA)  Mother’s current cigarette smoking status  Other adult smokers in the home  Marital status  Complete home smoking ban |
| Hawkins & Berkman, 2013[[35](#_ENREF_35)]  USA  Quality rating:  5 | Population-based cross sectional survey  2000-2003 | Pregnancy Risk Assessment Monitoring System (PRAMS) data.  Used data from 2000-2003.  Response rate not reported. | N = 135278 mothers  Approximately 4 months of age | Parental reported child exposure:  ‘about how many hours a day, on average, is your new baby in the same room with someone who is smoking?’ (coded 0 or 1+) | Number of children in household*  Maternal race/ethnicity*  Maternal education (years)*  Maternal age*  Marital status*  On WIC during pregnancy* | Chi square test  Multiple logistic regression | Exposure in household (Similar sig. associations found for 2 analyses: mother a current smoker/mother non-smoker)  Number of children in household  Maternal race/ethnicity  Maternal education  Maternal age  Marital status  On WIC during pregnancy |
| Hughes et al. 2008[[48](#_ENREF_48)]  Korea  Quality rating:  6 | Cross sectional  2002 | Random sampling of residential telephone numbers | N = 207  <18 years of age | Based on child whom respondent thought had the highest exposure.  Respondent reported number of cigarettes child exposed to per week at home and in other locations. | Respondent gender  Respondent age*  Marital status (married, not married)  Job class (at home, white collar, blue collar)  Education (≤ high school, ≥ college)  Cigarette smoking status*  Spouse cigarette smoking status*  Respondent’s parental cigarette smoking status*  Respondent friend’s cigarette smoking status  Smoking policy in the home (allowed, not allowed)*  Children <6 years in household  Number of anti-secondhand smoke message sources aware of  Number of groups discouraging smoking aware of  Spouse discourages smoking  Siblings discourage smoking  Confidence in protecting child from SHS (low/medium, high) | Chi-square test  Multiple logistic regression  Initial multivariate model included all variables that reached p < 0.15 significance in bivariate analysis. | Respondent/spouse being a current smoker  Respondent’s parent’s smoke  Home smoking ban |
| Jarvis et al. 1992[[30](#_ENREF_30)]  Scotland  Quality rating:  7 | Cross sectional  September 1986 | One third of primary schools in Edinburgh, Scotland chosen at random, and parents of all children in grade three contacted by postal questionnaire.  Response rate: 67% | N = 734  6-7 years of age | Salivary cotinine | Number of smokers in household*  Home ownership*  Single parent household*  Social class (British Registrar General’s classification)*  Month of examination*  Number of children in household*  Crowding (persons per room)*  Gender*  Day of examination* | Log transformed salivary cotinine used  Multiple linear regression | Number of smokers in household  Home ownership  Single parent household  Social class (British Registrar General’s classification)  Month of examination  Number of children in household  Crowding (persons per room)  Gender  Day of examination |
| Johansson et al. 2004[[50](#_ENREF_50)]  Sweden  Quality rating:  9 | Cross sectional cohort survey  April 2001 – January 2003 | All Babies in South East Sweden (ABIS).  Cohort comprises 17055 (78.6%) of the children born in the South East region of Sweden between Oct 1997 – Sept 1999.  Response rate 84% (n = 578) responded  Urine sample was received from 63.3% of these (n = 366). These were age-matched with controls from non-smoking cohort members (n = 433) | N = 799366 ETS exposed.  2.5-3 years of age | Urinary cotinine (above or below quantification level, 6 ng/ml)  Parental reported smoking in the home:  -Outdoors  -Open door and outdoors  -Kitchen fan and outdoors  -Mixers (smoked close to the kitchen fan or near an open door, or outdoors with the door closed.  -Indoor smoking  Dependent variables dichotomized as smoking indoors or outdoors, and urine CCR as above or below quantification level (6 ng/ml) | Cigarettes per day*  Family situation (nuclear/broken)*  Ethnicity*  Which parent smokes*  Exposure outside of home*  Size of dwelling* | Mann-Whitney U test.  Spearman’s correlation  Multiple logistic regression | Family situation (broken home)  Smoking behaviour  Cigarettes per day |
| Jurado et al. 2004[[54](#_ENREF_54)]  Spain  Quality rating:  8 | Cross sectional  April – May 1999 | 2 stage cluster sampling of 25 primary schools, and children within those schools  Response rate = 69.3% | N = 115  3-6 years of age | Urinary cotinine | Child age  Child gender  Paternal education (primary, secondary, technical, university)*  Maternal education (primary, secondary, technical, university)*  Index of crowding*  Day of week urine sample collected*  Number of cigarettes smoked at home  Paternal cigarette smoking status  Maternal cigarette smoking status*  Number of smoking parents*  Location of parental smoking in the home  Parental perception of smokiness at home* | Log transformed urinary cotinine used  ANOVA  Multiple linear regression | Paternal education  Day of week sample collected  Parental perception of smokiness at home |
| Liao et al.2013[[61](#_ENREF_61)]  Taiwan  Quality rating:  5 | Cross sectional survey  2010 | Quota sampling to divide counties and cities of Taiwan into 4 regions, according to their level of urbanisation and access to resources.  5 primary schools, and 2 classes selected at random at each school, which were than randomly assigned to cluster invite either fathers or mothers of the students to participate in study.  Current smokers included in analysis (smoked more than 100 cigarettes in their lifetime and smoking on more than one day during the preceding month).  Response rate 86%. | N = 307  Primary schools, grade 1-6 (ages 6 – 12 years of age) | Parental reported home smoking bans (dichotomous variables):  ‘do you have smoking bans at home?’  ‘does your family consistently enforce smoking bans at home?’ | Demographic characteristics:  Gender  Parental Age  Marital status  Parent who smoked education (qualification)*  Occupation  Family type (nuclear/mixed)  Annual income*  Grades of children  Region of Taiwan  Smoking variables:  Daily smoker*  Cigarettes per day*  Age smoked first cigarette  Ever considered quitting*  Attempting to quit in preceding year  Advised to quit by health care professional  Agreed with home smoking bans*  Had smoking bans at home*  Enforcement of smoking bans at home  Perceptions of smoking in the presence of their children*  Evaluations of the consequences of smoking in the presence of children*  Family’s anti-smoking responses to parental smoking in the presence of children*  Smoker’s reaction to family’s antismoking responses* | Chi square test  Hierarchical logistic regression models | Agreed with home smoking bans  Had smoking bans at home |
| Longman & Passey, 2013[[56](#_ENREF_56)]  Australia  Quality rating:  6 | Cross sectional survey  April – Sept 2010 | National drug strategy Household Survey, conducted every 3 years by the Australian institute of Health and Welfare.  Multistage random sample of households stratified by region with some oversampling in certain states and territories.  Response rate not reported. | N = 4669 households  <15 years of age | Parental reported home secondhand smoke exposure: ‘in the last 12 months, have you or any other member of your household smoked at least one cigarette, cigar or pipe of tobacco per day in the home?’  1) yes, smokes inside the home, 2) no does not smoke inside the home (no, only smokes outside the home, or no-one at home regularly smokes). | Rurality*  Socioeconomic status (area level socioeconomic index – SEIFA, based on income, education, employment, occupation and housing)*  Household size*  Household structure* | Chi square test  Multiple logistic regression. All variables with p < 0.25 in univariate analyses were included in the models, with stepwise removal of variables with variables with p < 0.1 retained in model | Rurality  Socioeconomic status  Household size  Household structure |
| Mannino et al. 2001[[39](#_ENREF_39)]  USA  Quality rating:  8 | Cross sectional survey  1988 – 1994 | Third National Health and Nutrition Examination Survey (NHANES III), conducted by the National Center for Health Statistics of the Centers for Disease Control and Prevention, Atlanta.  Stratified, multistage, clustered probability design to select representative sample of US population.  Limited analysis to children aged 4-16 years with valid serum cotinine levels. | N = 5653  4-16 years of age | Serum cotinine | Child age*  Child gender*  Region*  Parental education*  Race/ethnicity*  Family poverty index (below or at poverty line, above poverty line, unknown)*  Family size (≤4, ≥5)*  Number of rooms  Number of cigarettes smoked in home* | Log transformed serum cotinine used.  Used sampling weights to account for non-response  Multiple linear regression | Child age  Parental educational level  Race/ethnicity  Number of rooms  Number of cigarettes smoked in the home |
| Mantziou et al. 2009[[45](#_ENREF_45)]  Greece  Quality rating:  7 | Cross sectional  September – December 2004 | Recruited from emergency departments of two paediatric hospitals in Athens.  Response rate = 100% | N = 662  <12 years of age | Parental reported child SHS exposure (smoking in the home in front of children) | Child gender*  Child age*  Paternal age  Maternal age  Number of smokers in the house  Number of children*  Paternal cigarettes per day*  Maternal cigarettes per day*  Child gender*  Housing (apartment building/freestanding)  Maternal education (lower/higher)*  Paternal education (lower/higher)*  Friends/relatives smoke at home*  Bothered if child became smoker* | T-test  Chi-square  Backward stepwise logistic regression model | Paternal cigarettes per day  Child age |
| Mills et al. 2012[[29](#_ENREF_29)]  Scotland  Quality rating:  7 | Intervention study  July 2010 – March 2011 | REFRESH intervention.  Potential participants identified through GP records by the Scottish Primary Care Research Network.  Response rate: 3.1% of invite letters sent. | N = 54  1-5 years of age | Airborne particulate matter  Salivary cotinine  Parental reported smoking restrictions:  1)not allowed inside the home, 2) child based restrictions, e.g. no smoking in a room when a child is present, 3) room based restrictions, 4) no restrictions  Restrictions on smoking in the car: 1) not allowed, 2) partial restrictions, e.g. no smoking if a child or non-smoker was present, 3) no restrictions, 4) no car | Number of cigarettes smoked at home by mother*  Child age*  Maternal age*  Child gender*  Scottish Index of multiple deprivation (SIMD)*  Accommodation type*  Number of smokers in household*  Maternal attitudes to SHS exposure  Household smoking restrictions*  Smoking restrictions in the car* | Skewed data log transformed  T test  Chi square test  Stepwise multiple linear regression analysis to identify factors associated with airborne particulate matter levels and saliva cotinine. | Air quality:  Number of cigarettes smoked at home  Salivary cotinine:  Child age  Maternal age  Household smoking restrictions  Maternal attitudes to SHS exposure |
| Moore et al. 2011[[31](#_ENREF_31)]  Wales  Quality rating:  8 | Repeated cross sectional survey  31^st^ January 2007 – 30^th^ March 2007  31^st^ January 2008 – 28^th^ April 2008 | CHETS Wales study (Changes in Child Exposure to Environmental Tobacco Smoke).[[99](#_ENREF_99)]  Recruited across 75 state primary schools in Wales.  Pre-legislation, 91.5% response rate, 82.2% valid cotinine samples.  Post legislation, 90.4% response rate, 82.3% valid cotinine samples. | Pre-legislation N = 1611 (91.5%) of students completed questionnaire  Post legislation N = 1605 (90.4%) completed questionnaire  Cotinine available:  Pre-legislation N = 1447 (82.2%)  Post legislation N = 1461 (82.3%)  10-11 years | Salivary cotinine  Child reported parental smoking in the home. Subsequently categorised depending on which parental figures smoked in the home (neither, father figure only, mother figure only or both)  Child reported SHS exposure in cars (response to question ‘while you were in the car yesterday, was anyone smoking there?’) | Socioeconomic status (Family Affluence Scale)  Analysis controlled for:  Age  Year of data collection  Time of data collection | Salivary cotinine levels divided into tertiles (low, <0.10 ng/ml; medium, 0.1-0.5 ng/ml and high, >0.5 ng/ml)  Multinomial regression analysis. | Family affluence  Interaction between FAS and survey year on child salivary cotinine  Interaction between SES and survey year on child reported parental smoking in the home |
| Peltzer, 2011[[59](#_ENREF_59)]  South Africa  Quality rating:  7 | Cross sectional  2008 | Global Youth Tobacco Survey  Two-stage cluster sample design; schools selected with probability proportional to enrolment size. Classes within these schools were then randomly selected.  Response rate 77.9% | N = 6412  11-18 years of age | Child reported exposure to SHS at home and SHS exposure:  ‘During the past 7 days, on how many days have people smoked in your home, in your presence?’  ‘During the last 7 days, on how many days have people smoked in your presence, in places other than your home?’ | Child gender*  Child age*  Parental cigarette smoking status*  Friends cigarette smoking status*  Child attitudes towards SHS exposure* | Univariate logistic regression  Multiple logistic regression | Parental cigarette smoking status  Friend cigarette smoking status  Child attitudes towards SHS exposure |
| Pisinger et al. 2012[[49](#_ENREF_49)]  Denmark  Quality rating:  3 | Cross sectional survey  2007 and 2010 | ‘Health profiles of the capital region’ survey of 2007 and 2010. Random samples of all citizens drawn from the civil registration system using random numbers.  Includes participants of the survey who completed the question ‘does smoking take place indoors in your home?’  Response rate 52.3% in both survey years. | 2007 N = 9289  2010 N = 12696  <15 years of age | Parental reported smoking in the home:  ‘does smoking take place indoors in your home?’ Dichotomised as 1) no (never or almost never/less than weekly), 2) yes (weekly/daily) | Respondent gender*  Age*  Daily smoker  Tobacco consumption  Education (very low, low, medium, high – taken as a measure of socioeconomic status) | Weighted for size of municipality and non-response  Multiple logistic regression | Education level |
| Preston et al. 2001[[64](#_ENREF_64)]  Puerto Rica  Quality rating:  7 | Cross sectional  August 1993 – November 1996 | Recruited at routine appointments at Paediatric Primary Care Clinic | N = 606  2-12 years of age | Urinary cotinine | Child age*  Child gender  Maternal age*  Maternal civil status (living with partner, living alone)*  Receiving food stamps  Maternal education (> 8^th^ grade, 0-8^th^ grade)*  Maternal employment (employed, unemployed and/or housewife)*  Season of year (summer, winter)* | Log transformed urinary cotinine was used.  F-test  Kruskal-Wallis tests  Multiple linear regression | Maternal civil status (living with partner,  living alone)  Receiving food stamps  Child age |
| Rachiotis et al. 2009[[46](#_ENREF_46)]  Greece  Quality rating:  7 | Cross Sectional  2004 – 2005 | Analysis of the Global Youth Tobacco Survey, Greece.  Two-stage cluster sampling. 25 schools from each region containing the middle-school grades in Greece selected. Classes within selected schools randomly sampled. | N = 5179  11-17 years of age | Child reported exposure to SHS at home and SHS exposure:  ‘How often do you see your father/mother/sister/friend/other people smoking in your home?’  Don’t have/don’t see this person; about every day; sometimes; never. | Child age*  Child gender*  Parental cigarette smoking status*  Friends cigarette smoking status* | Multiple logistic regression | Child age  Child gender  Parental cigarette smoking status  Friends cigarette smoking status |
| Raisamo et al. 2013[[51](#_ENREF_51)]  Finland  Quality rating:  5 | Repeated cross sectional survey  1991-2009 | Adolescent health and lifestyle survey’, conducted biennially.  Population register sampled on the basis of particular dates of birth.  Response rate ranged from 77% (1991) to 56% (2009). | N = 72726  12-18 years of age | Child reported daily exposure to SHS: ‘about how many hours a day do you spend in rooms where people smoke?’ Dichotomised as 1) exposed to environmental tobacco smoke < 1 hour per day, 2) exposed to environmental tobacco smoke for an average of 1 hour a day or more. | Paternal education (high, middle, low)  Maternal education (high, middle, low)  Family structure (intact family/other)  Child’s School performance  Child’s School attended  Parental cigarette smoking status  Analysis controlled for:  Age  Gender  Study period | Multiple logistic regression | Parental smoking |
| Raute et al. 2012[[60](#_ENREF_60)]  India  Quality rating:  6 | Cross sectional  July – September 2010 | Mumbai Student Tobacco Survey  Two stage cluster sampling design across 26 schools in Mumbai region. | N = 1511  11-17 years of age | Child reported SHS exposure: ‘during the past seven days, on how many days have people smoked in your home, and in your presence?’ | Child tobacco use*  Child age*  Gender*  Parent’s tobacco use*  Close friends smokers*  Awareness about current ban in public places*  Awareness about harmfulness of exposure to SHS from other people*  Transport to school* | Multiple logistic regression | Child tobacco use  Parents smokers  Close friends smokers  Awareness about harmfulness of exposure to SHS from other people  Transport to school |
| Ren et al. 2012[[40](#_ENREF_40)]  USA  Quality rating:  2 | Cross Sectional  October 2006 – March 2008 | Recruited in General Paediatric Clinic at Children’s Hospital of Michigan in Detroit. Mothers accompanying one or more children aged 7-10 years eligible.  Response rate 80% | N = 399  7 – 10 years | Maternal reported child SHS exposure:  ‘During the past year, how many smokers lived in or frequently visited your home?’  ‘How many of them are daily smokers?’  ‘Among these daily smokers, how many smoked when the (index child) was around?’  Child defined as exposed to SHS if exposed to at least one daily smoker in previous year. | Pregnancy unplanned  Maternal education (≤ High school, ≥ college)*  Marital status (married/not married)  Parenting satisfaction (strongly satisfied, satisfied, dissatisfied)*  Number of children (1-2, 3-4, 5+)  Monthly family income ($200-$1000; $1001 - $2000; $2001 - $3000; $3001+) | Chi square test  Multiple logistic regression | Among non-smoking mothers:  Pregnancy unplanned  Maternal education |
| Rise & Lund, 2005[[52](#_ENREF_52)]  Norway  Quality rating:  3 | Cross sectional  May 1995 and August 2001 | Postal survey sent to stratified random sample of 1000 households with 3 year old children, drawn from Central Office of Population Records. | 1995 N = 212  2001 N = 2001  3 years of age | Parental reported child SHS exposure: ‘is your child present when someone smokes – in the car, sleeping room, TV-room, dining rooms, elsewhere at home’. | Household education (years)  Awareness of smoking risks  Attitudes towards SHS | Regression | Education  Attitudes towards ETS |
| Rudatsikira et al. 2007[[58](#_ENREF_58)]  Mongolia  Quality rating:  5 | Cross sectional  2003 | Global Youth Tobacco Survey  Two stage cluster sampling; schools selected with probability proportional to enrolment size, and classes within these schools randomly selected. | N = 3507  13-15 years of age | Child self-reported SHS exposure: had people smoke in their presence on one or more days in the previous 7 days (both at home or outside of the home) | Child age  Child gender  Parental smoking  Friends smoking  Confounders controlled for – not known | Logistic regression (not known if multiple logistic regression conducted) | Child age  Parents smoking  Friends smoking |
| Scherer et al. 2004[[42](#_ENREF_42)]  Germany  Quality rating:  4 | Cross sectional  1996 – 1998 | All school-entrance children in Augsburg, Southern Germany, invited to take part in Multicentric International Study for Risk Assessment of Indoor and Outdoor Air on Allergy and Eczema Morbidity (MIRIAM).  Children who had valid urine sample for both 1996-1998 were eligible for inclusion. | N = 258  6-7 years of age | Urinary cotinine | Parental smoking  Number of smokers in household  Cigarettes per day smoked in home  Child gender  Nationality  Parental education (elementary school or less, intermediate high school, high school, University)  Bedroom sharing  Size of flat  Leisure time activity (preferred place of stay during free time, regularly exercising, free time spent watching TV) | Log transformed urinary cotinine used.  Linear regression | Parental smoking  Number of smokers in household  Cigarettes per day smoked in home  Nationality  Bedroom sharing  Size of flat  Leisure time activity (regularly exercising, free time spent watching TV) |
| Sims et al. 2010[[5](#_ENREF_5)]  England  Quality rating:  8 | Repeated cross sectional survey  1996 to 2006 | Health survey for England, 1996-2006 (excluding 1999, 2000 and 2004 when cotinine samples were not available).  70.1% of sample returned valid salivary cotinine sample (83.5% in 1996, 58.3% in 2006). | N = 9289 children   4-15 years of age | Salivary cotinine  Parental cigarette smoking status (positive response to questions ‘do you smoke cigarettes at all nowadays?’)  SHS exposure (positive response to 2 questions: whether ‘someone smokes inside the home most days’, whether ‘children were looked after for more than 2 hours per week by someone who smokes whilst looking after them’) | Study year*  Child age*  Child gender*  Social class of head of household, Registrar General’s Social Class(I, professional; II managerial and technical; III skilled non-manual and manual; IV semi-skilled manual; V unskilled manual)*  Head of household employment status*  Education status of parents (highest qualification of either parent)  Crowding (people per bedroom)  Ethnicity*  Parental cigarette smoking status*  Smoking in the home*  Carer smoking (> 2 hours per week)* | Log transformed salivary cotinine used.  Child salivary cotinine: Linear regression, adjusted for survey year and age.  Multivariate linear regression: Backward selection procedure using mean log cotinine | Year (pre/post legislation)  Child age  Gender (only in 4-12 year olds)  Social class  Employment status  Education  Ethnicity  Parental cigarette smoking status  Smoking in the home  Crowding (people per bedroom) |
| Singh et al. 2010[[38](#_ENREF_38)]  USA  Quality rating:  5 | Cross sectional  April 2007 – July 2008 | National Survey of Children’s Health & Current Population Survey – Tobacco Use Supplement  Random digit dial survey | N = 90853  ≤17 years of age | Parental reported smoking in the home: ‘does anyone in your household use cigarettes, cigars or pipe tobacco?’ ‘Does anyone smoke inside child’s home?’ | USA State*  Child age*  Child gender*  Race/ethnicity*  Household composition (two parent biological, two parent step family, single mother, other family type)*  Place of residence (metropolitan, non-metropolitan)*  Primary language spoken at home (English, other)*  Household poverty status*  Highest household/parental education level* | Chi square test  Multiple logistic regression | USA state  Race/ethnicity  Household composition  Place of residence (metropolitan/non metropolitan)  Primary language spoken at home  Household poverty status  Highest household/parental education level |
| Soliman et al. 2004[[33](#_ENREF_33)]  USA  Quality rating:  4 | Repeated cross sectional survey  1992 and 2000 | 1992 and 2000 National Health Interview Survey (NHIS).  Multistage area probability sampling design.  Response rate not reported. | 15,601 families   ≤18 years of age | Number of days per week someone smoked in the home. | Region*  Race/ethnicity*  Maternal education (qualification)*  Attitudes towards SHS (SHS harmful, not harmful, unsure)* | Multiple Logistic regression | Survey year  Region  Race/ethnicity  Maternal education  Attitudes towards SHS |
| Ulbricht et al. 2014[[43](#_ENREF_43)]  Germany  Quality rating:  6 | Cross sectional  Up to January 2008 | Recruited as part of home brief intervention trial.  Recruited in rural region in German Federal State of Mecklenburg – West Pomerania.  Self-reported currently smoking households with a child aged three years or younger included in sample.  71.5% response rate. | N = 917  ≤ 3 years of age | Respondent reported indoor smoking in homes: ‘where in the private area of the household is smoking allowed?’ 1) nowhere, 2) on balcony/terrace, 3) in specific rooms only, 4) everywhere.  Smoking in home defined as smoking in specific rooms only or everywhere at home. | Nursery attendance by target child*  Presence of balcony/terrace/garden*  Household crowding (number of people per room: less than one person, one person, more than one person)*  Number of children in household*  Household highest education level. Low (secondary school certificate or no graduation), middle (intermediate general school certificate), high (qualification for university entrance)*  Household employment (noun employment, part unemployment, full unemployment)*  Household/parental cigarette smoking status*  Number of respondent’s two closest friends who smoked*  *Controlled for child age in multivariate analysis | Univariate logistic regression  Multivariate logistic regression | Nursery attendance by target child  Presence of balcony/terrace/garden  Household crowding  Number of children in household  Household highest education level.  Household employment  Household/parental cigarette smoking status  Number of respondent’s two closest friends who smoked |
| Whitrow et al. 2010[[26](#_ENREF_26)]  UK  Quality rating:  7 | Cross sectional  2003-2004 | Sample recruited from 51 schools in 10 inner London Boroughs with high proportions of the main ethnic minority groups. All pupils aged 11-13 years in randomly selected mixed ability classes invited to take part.  Response rate 81% | N = 3468  11-13 years of age | Salivary cotinine | Ethnicity (White, black Caribbean, black African, Indian, Pakistani, Bangladeshi)  Adjusted for age, sex, day of week sample taken | Log transformed salivary cotinine used  Multiple linear regression | Ethnicity |
| Yi 2012[[47](#_ENREF_47)]  Korea  Quality rating:  7 | Cross sectional cohort survey  2008 | Children’s health and environmental research (CHEER).^[^[^100^](#_ENREF_100)^]^  Parents of school-aged children from 33 schools in 10 representative cities invited to participate.  Response rate not reported. | N = 7059 children  6-10 years of age | Parental report child exposure: ‘has your child ever been exposed to smoke from tobacco in the household?’  Urinary cotinine | Child gender^  Marital status*^  Family size*^  Type of accommodation*^  Maternal education (years)*^  Paternal education (years)*^  Household income*^  Parental reported SHS exposure^  Deprivation (Carstairs index measuring area-based deprivation, using low social class, lack of car ownership, overcrowding and male unemployment to categorise geographical areas)*^  Parental SES and deprivation interaction  *controlled for in parental reported SHS exposure analysis  ^controlled for in child cotinine level analysis | Log transformed urinary cotinine used.  Simple logistic regression  Multiple logistic regression | Parental reported child SHS exposure:  Type of accommodation  Maternal education  Paternal education  Household income  Deprivation  Deprivation X paternal education interaction  Child serum cotinine levels:  Deprivation area  Environmental tobacco smoke  Type of accommodation |

**ˆ** Quality assessment using the modified Newcastle-Ottawa Quality Assessment Scale,[[19](#_ENREF_19),[20](#_ENREF_20)] maximum score 10, higher score reflecting higher quality

** Significance level used in individual studies taken to be p < 0.05 unless otherwise stated

Additional references

98. Akhtar PC, Currie DB, Currie CE, Haw SJ (2007) Changes in child exposure to environmental tobacco smoke (CHETS) study after implementation of smoke-free legislation in Scotland: national cross sectional survey. BMJ 335: 545.

99. Holliday JC, Moore GF, Moore LAR (2009) Changes in child exposure to secondhand smoke after implementation of smoke-free legislation in Wales: a repeated cross-sectional study. Bmc Public Health 9.

100. Ha M, Kwon H-J, Lim M-H, Jee Y-K, Hong Y-C, et al. (2009) Low blood levels of lead and mercury and symptoms of attention deficit hyperactivity in children: a report of the children's health and environment research (CHEER). Neurotoxicology 30: 31-36.
