# Supplementary material for: Predictors of Children's Secondhand Smoke Exposure at Home: A Systematic Review and Narrative Synthesis of the Evidence
Source: PLoS One. 2014 Nov 14;9(11):e112690. doi: 10.1371/journal.pone.0112690 (PMC4232519; doi:10.1371/journal.pone.0112690)
Supplement: Table S2 — Associations identified and strength of effect. (DOCX) [file pone.0112690.s002.docx]

| Study | Association measurement | Outcome measure | Adjusted effect size († Univariate analysis only) |
| --- | --- | --- | --- |
| *Socioeconomic status* | | | |
| Cook et al.[[32](#_ENREF_32)] | Registrar General’s Social Class system | A | T Test for trend = 11.5, p = 0.0001 (trend for higher geometric mean cotinine with decreasing social class) |
| Delpisheh et al.[[25](#_ENREF_25)] | Townsend score | A | Townsend score > +6 OR = 1.2 (95% CI 1.0-1.4) |
| Jarvis et al.[[30](#_ENREF_30)] | Registrar General’s Social Class system | A | Β = 1.19 (95% CI 1.11-1.27) (lower social class experience more exposure) |
| Moore et al.[[31](#_ENREF_31)] | Family Affluence Scale (FAS) | A | Risk ratio (RR) of a child’s sample containing low level of cotinine (< 0.10 ng/ml) RR =1.16 (95% CI 1.10-1.22)  RR of sample containing high level of cotinine (> 0.50 ng/ml) RR = 0.82 (95% CI 0.77-0.88)  RR of Child providing a saliva sample with a low cotinine sample (< 0.10 ng/ml) post legislation:  Low SES households, ref p > 0.05  Medium SES households RR = 1.66 (95% CI 1.20-2.3)  High SES households and RR = 1.44 (95% CI 1.04-2.0) |
| Sims et al.[[5](#_ENREF_5)] | Registrar General’s Social Class system | A | 4-15 year olds  I and II (professional, managerial and technical) – (ref)  III (skilled non-manual and manual) β 0.133 (95% CI 0.084 – 0.181)  IV and V (semi-skilled and unskilled manual) β 0.253 (95% CI 0.189-0.316) |
| Akhtar et al.[[27](#_ENREF_27)] | Family Affluence Scale (FAS) | A | Low β 0.41 (95% CI 0.29 – 0.5)  Medium β 0.08 (95% CI -0.5 – 0.2)  High – ref |
| Akhtar et al.[[28](#_ENREF_28)] | Registrar General’s Social Class classification system  Family Affluence Scale (FAS) | A | Family SEC (ref: SEC 1):  SEC 2 β 0.32 (95% CI 0.17 to 0.47) p <0.001  SEC 3 β 0.45 (95% CI 0.26 to 0.65)  SEC 4 β 0.82 (95% CI 0.60, 1.04)  Family affluence scale (FAS) (ref: high FAS):  Medium FAS β 0.15 (95% CI 0.01 to 0.29) p <0.001  Low FAS β 0.41 (95% CI 0.27 to 0.55) |
| Baheiraei et al.[[63](#_ENREF_63)] | Registrar General Model of Social Class | C | Employer, junior employee or lower OR 9.84 (95% CI 2.33 – 41.46)  Skilled workers OR 2.14 (95% CI 0.8 – 5.73)  Semi-skilled or unskilled workers – ref |
| Yi et al.[[47](#_ENREF_47)] | Area level deprivation | E | Most deprived (>75%) OR 1.34 (95% CI 1.06,1.69)  25%–75% OR 1.19 (95% CI 1.02,1.39)  Least deprived (<25%), Ref. |
| Longman & Passey[[56](#_ENREF_56)] | Area-level socioeconomic index | E | 1 (lowest), ref  2,OR 0.55 (95% CI 0.41 to 0.74)  3, OR 0.52 (95% CI 0.38 to 0.70)  4, OR 0.27 (95% CI 0.19 to 0.38)  5 (highest), OR 0.25 (95% CI 0.17 to 0.37) |
| *Income* | | | |
| Chen et al.[[37](#_ENREF_37)] | Household income | B | ≤ $2500 OR 2.32 (95% CI 1.47 – 3.68)  ≥ $ 2501 – ref |
| Bolte & Fromme[[41](#_ENREF_41)] | Household equivalent | E | >median, ref  60% of median-media, OR 1.29 (95% CI 1.13–1.46)  <60% median (relative poverty), OR 1.45 (95% CI 1.21–1.74)  Not indicated/refused, OR 0.92 (95% CI 0.82–1.03) |
| Singh et al.[[38](#_ENREF_38)] | Household poverty status (ratio of family income to federal poverty guidelines) | E | < 100% OR 3.02 (95% CI 2.41 – 3.78)  100% - 199% OR 2.61 (95% CI 2.1 – 3.24)  200% - 399% OR 1.86 (95% CI 1.52 – 2.28)  ≥ 400% - ref |
| Yi et al’s.[[47](#_ENREF_47)] | Combined household annual | E | <$1,800, OR 1.28 (95% CI 1.10,1.49)  $1,800–2,700, OR 1.08 (95% CI 0.95,1.24)  ≥$2,700, ref. |
| *Employment status* |  |  |  |
| Sims et al.[[5](#_ENREF_5)] | Head of household | A | 4-15 year olds:  Employed (ref)  Unemployed β 0.914 (95% CI 0.810-1.018)  Other β 0.914 (95% CI 0.839-0.990) |
| Bolte& Fromme[[41](#_ENREF_41)] | Parental employment | E | Parental reported child SHS exposure in home  At least one parent employed, ref  Both parents only marginally employed or unemployed, OR 1.88 (95% CI 1.60–2.21) |
| Ulbricht et al.[[43](#_ENREF_43)] | Household employment | E | No employment – ref  Partial employment (one parent works), OR 2.38 (95% CI 1.54 – 3.68), p = 0.01 |
| *Occupation* | | | |
| Abidin et al.[[57](#_ENREF_57)] | Paternal occupation (armed forces, manager/professional) | A | Manager/professional – ref  Armed forces standard β = 0.16, p < 0.0001 |
| *Other socioeconomic* | | | |
| Jarvis et al.[[30](#_ENREF_30)] | Home ownership | A | Own home β = 1.42 (95% CI 1.16 – 1.72) |
| Hawkins & Berkman[[35](#_ENREF_35)] | WIC use during pregnancy | F | Smoking mothers:  WIC during pregnancy OR 1.41 (95% CI 1.26, 1.57) |
| *Parental characteristics* | | | |
| *Parental education* | | | |
| Abidin et al.[[57](#_ENREF_57)] | Paternal education (diploma/technical certificate, degree/college) | A | Degree/college – ref  Diploma/technical certificate standard β = 0.08, p = 0.021 |
| Sims et al.[[5](#_ENREF_5)] | Highest qualification of either parent | A | 4-15 year olds:  Higher education qualification – ref  School level (or other) β 0.665 (95% CI 0.613-0.717)  No qualifications β 1.308 (95% CI 1.227-1.390) |
| Chen et al.[[37](#_ENREF_37)] | Maternal | B | ≤ High school OR 2.32 (95% CI 1.47 – 3.  >High school – ref |
| Dell’Orco et al.[[53](#_ENREF_53)] | Paternal education (years) | B | >13 years – ref  9-13 ratio 1.24 (95% CI 1.01-1.52)  6-8 ratio 1.38 (95% CI 1.13 – 1.68)  <6 ratio 1.34 (95% CI 1.09 – 1.64)  Unknown ratio 1.31 (95% CI 0.86 – 2.01) |
| Jurado et al.[[54](#_ENREF_54)] | Paternal education (primary, secondary, technical, university) | B | r-partial -0.208 (p = 0.05) Higher education associated with lower child cotinine |
| Mannino et al.[[39](#_ENREF_39)] | Parental education (years) | C | Years, mean increase in log cotinine, ng/ml  < 12 or unknown – 0.39 (95% CI 0.21 – 0.58)  12 – 0.32 (95% CI 0.2 – 0.44)  > 12 – ref |
| Anuntaseree et al.[[62](#_ENREF_62)] | Paternal education (primary, secondary, college/university) | E | Primary OR 2.1 (95% CI 1.5 – 3.0)  Secondary OR 1.7 (95% CI 1.2 – 2.5)  College or university - ref |
| Bolte & Fromme[[41](#_ENREF_41)] | Parental education (very high, high, middle, low) | E | Very high – ref  High, OR 2.52 (95% CI 2.18–2.93)  Middle, OR 2.37 (95% CI 2.09–2.68)  Low, OR 3.94 (95% CI 3.46–4.49) |
| Pisinger et al.[[49](#_ENREF_49)] | Respondent education (very low, low, medium, high) | E | 2010 (95% CI not reported)  High – ref  Medium OR 2.2  Low OR 4.6  Very low OR 10.4 |
| Ren et al.[[40](#_ENREF_40)] | Maternal education (≤ high school, ≥ college) | E | Mothers who did not smoke:  P < 0.01 |
| Rise & Lund[[52](#_ENREF_52)] | Household education (years) | E | 1995: Β = 0.17, p < 0.05  2001: β = 0.16, p < 0.05 |
| Singh et al.[[38](#_ENREF_38)] | Highest household education (years) | E | <12 OR 3.56 (95% CI 2.72 – 4.66)  12 OR 2.93 (95% CI 2.41 – 3.56)  13-15 OR 2.32 (95% CI 1.92 – 2.81)  ≥16 – ref |
| Soliman et al.[[33](#_ENREF_33)] | Maternal qualification | E | High school dropout OR 1.18 (95% CI 1.03-1.35)  High school graduate – ref  Some college OR 0.64 (95% CI 0.57-0.71)  College OR 0.36 (95% CI 0.3-0.43)  Postgraduate OR 0.28 (95% CI 0.21-0.37) |
| Ulbricht et al.[[43](#_ENREF_43)] | Highest household | E | Low – ref  Middle OR 0.52 (95% CI 0.32 – 0.86), p = 0.01  High OR 0.30 (95% CI 0.17 – 0.54), p < 0.0001 |
| Yi et al.[[47](#_ENREF_47)] | Maternal and paternal (years) | E | Maternal:  <12 years, OR 1.23 (95% CI 0.88,1.72)  12 years, OR 1.28 (95% CI 1.12,1.47)  >12 years – ref.  Paternal:  <12 years, OR 1.81 (95% CI 1.30, 2.54)  12 years, OR 1.42 (95% CI 1.24, 1.63)  >12 years – ref. |
| Hawkins & Berkman[[35](#_ENREF_35)] | Maternal education, years | F | Smoking mothers:  0–11 ref  12 OR 1.00 (95% CI 0.88, 1.12)  13–15 OR 0.80 (95% CI 0.68, 0.95)  16+ OR 0.59 (95% CI 0.45, 0.77) |
| Raisamo et al.[[51](#_ENREF_51)] | Maternal and paternal education (high, middle, low) | F | 12–14-year-olds:  Paternal education  High – (ref)  Middle OR 1.6 (95% CI 1.1–2.8)  Low OR 2.3 (95% CI 1.1–2.8)  Maternal education  High – (ref)  Middle OR 1.4 (95% CI 1.1–1.9)  Low OR 2.4 (95% CI 1.1–3.1)  16-18 year olds  Paternal education  High – (ref)  Middle OR 1.3 (95% CI 1.1 – 1.4)  Low OR 1.7 (95% CI 1.1 – 1.9)  Maternal education  High – (ref)  Middle OR 1.4 (95% CI 1.1–1.6)  Low OR 1.9 (95% CI 1.1 – 2.1) |
| Alwan et al.[[24](#_ENREF_24)] | Head of household qualification | E | Qualification below A-level OR 2.20 (95% CI 1.08-4.47) p = 0.03 |
| Liao et al.[[61](#_ENREF_61)] | Parent who smoked, qualification | E | ≥Baccalaureate – ref  High School 1.97 (95% CI 1.16-3.33)  Junior high 2.44 (95% CI 1.14-5.25) |
| *Parental age* | | | |
| Mills et al.[[29](#_ENREF_29)] |  | A | β = .0284, p < 0.05 |
| Anuntaseree et al.[[62](#_ENREF_62)] |  | E | Paternal age:  15-24 years – ref  25 – 34 years OR 1.6 (95% CI 1.2 – 2.2)  35 – 44 years OR 1.3 (95% CI 0.9 – 1.9)  >44 years OR 2.3 (95% CI 1.1 – 4.6) |
| Hawkins & Berkman[[35](#_ENREF_35)] |  | F | Smoking mothers:  <17–19 – ref  20–24 OR 0.94 (95% CI 0.82, 1.09)  25–29 OR 0.83 (95% CI 0.71, 0.98)  30–34 OR 0.79 (95% CI 0.66, 0.96)  35+ OR 0.82 (95% CI 0.65, 1.03) |
| *Race/ethnicity* | | | |
| Sims et al.[[5](#_ENREF_5)] |  | A | 4-15 year olds:  White – ref  Black/Asian β -0.183 (95% CI -0.260-0.105) |
| Scherer et al.[[42](#_ENREF_42)] |  | B | †  Nationality  % explained by variance (R^2^) = 4.6, p < 0.001  German geometric mean = 27.2  Non-German geometric mean = 34.1 |
| Whitrow et al.[[26](#_ENREF_26)] |  | B | Cotinine ng/ml (95% CI), p  White UK – (ref) 0.71 (95% CI 0.62 – 0.82)  Black Caribbean 0.3 (95% CI 0.44 – 0.36), p < 0.05  Black African 0.29 (95% CI 0.26 – 0.33), p < 0.05  Indian 0.27 (95% CI 0.23 – 0.31), p < 0.05  Pakistani 0.32 (95% CI 0.27 – 0.37), p < 0.05  Bangladeshi 0.5 (95% CI 0.41 – 0.6), p < 0.05 |
| Mannino et al.[[39](#_ENREF_39)] |  | C | Mean increase in log cotinine, ng/ml  White – ref  Black 0.10 (95% CI -0.06 – 0.26)  Mexican-American -0.73 (95% CI -0.93 - -0.53)  Other -0.29 (95% CI -0.61 – 0.03) |
| Bleakley et al.[[36](#_ENREF_36)] |  | E | White – ref  Black OR 7.08 (95% CI 2.92-17.16)  Other OR 3.82 (95% CI 1.05 – 14.02) |
| Singh et al.[[38](#_ENREF_38)] |  | E | Hispanic – ref  Non-Hispanic white OR 2.02 (95% CI 1.47-2.76)  Non-Hispanic black OR 3.63 (95% CI 2.6 – 5.09)  American Indian OR 2.0 (95% CI 1.24 – 3.25)  Asian OR 1.85 (95% CI 0.92 – 3.75)  Hawaiian/Pacific Islander OR 0.35 (95% CI 0.16 – 0.79)  Non-Hispanic mixed race OR 2.45 (95% CI 1.66 – 3.63)  Other OR 3.09 (95% CI 1.55 – 6.14) |
| Soliman et al.[[33](#_ENREF_33)] |  | E | White – ref  Hispanic OR 0.36 (95% CI 0.32-0.42)  African American OR 0.74 (95% CI 0.65-0.84)  Native American OR 1.12 (95% CI 0.74-1.69)  Asian OR 0.57 (95% CI 0.41-0.8) |
| Hawkins & Berkman[[35](#_ENREF_35)] |  | F | Smoking mothers:  White – (ref)  Hispanic OR 0.34 (95% CI 0.26, 0.45)  Black OR 0.67 (95% CI 0.58, 0.78)  Other OR 0.54 (95% CI 0.42, 0.70) |
| Anuntaseree et al.[[62](#_ENREF_62)] | Religion | E | Buddhist – ref  Muslim OR 6.7 (95% CI 4.8 – 9.4)  Christian OR 1.2 (95% CI 0.7 – 20.8)  Other OR 1.5 (95% CI 0.5 – 4.5) |
| *Parenting* | | | |
| Chen et al.[[37](#_ENREF_37)] | Parental satisfaction | B | Satisfied OR 0.57 (95% CI 0.36 – 0.91)  Not satisfied – ref |
| Ren et al.[[40](#_ENREF_40)] | Pregnancy unplanned | E | Non-smoking mothers:  Unplanned children more likely to be exposed to SHS in the home, p < 0.05 |
| *Parental smoking behaviour and attitudes* | | | |
| *Parental cigarette smoking status/number of smokers in household* | | | |
| Akhtar et al.[[27](#_ENREF_27)] |  | A | At least one parent a smoker:  Coefficient 1.2 (95% CI 0.99 – 1.42), p < 0.001 |
| Delpisheh et al.[[25](#_ENREF_25)] |  | A | Maternal cigarette smoking OR 2.5 (95% CI 1.8-3.4)  Presence of a smoker in the household OR 2/3 (95% CI 1.2-4.4) |
| Jarvis et al.[[30](#_ENREF_30)] |  | A | Smokers in household β = 3.57 (95% CI 3.2 – 3.98) (Exposure higher in households with more smokers) |
| Sims et al.[[5](#_ENREF_5)] |  | A | 4 – 15 year olds  Parental cigarette smoking status:  Neither parent smokes – ref  Father only smokes regression coefficient = 0.3 (95% CI 0.2 – 0.39)  Mother only smokes regression coefficient = 0.74 (95% CI 0.6 – 0.8)  Both parents smoke regression coefficient = 1.08 (95% CI 0.99 – 1.17) |
| Abidin et al.[[57](#_ENREF_57)] |  | A | Father only smoker std. beta 0.15, p < 0.0001  Father and family smoker std beta 0.09, p = 0.01  Neither parents smoke |
| Dell’Ocro et al.[[53](#_ENREF_53)] |  | B | Other smokers in household (in addition to parental smoking)  No – ref  Yes – ratio = 1.4 (95% CI 1.18 – 1.67)  Unknown – ratio = 0.97 (95% CI 0.78 – 1.21) |
| Scherer et al.[[42](#_ENREF_42)] |  | B | †  Parental cigarette smoking status: % variance explained (R^2^) 39.5, p < 0.001  Geometric mean urinary cotinine  None 10.3  Father only 29.1  Mother only 50.2  Both 48.4  Number of smokers in household: % variance explained (R^2^) 38.9, p < 0.001  0, geometric mean urinary cotinine = 10.2  1, geometric mean urinary cotinine = 34.4  2, geometric mean urinary cotinine = 45.4  3, geometric mean urinary cotinine = 56.6 |
| Gonzales et al.[[34](#_ENREF_34)] |  | E | Mother’s current cigarette smoking status  Non-smoker – ref  Smoker OR = 3.31 (95% CI 1.47 – 7.46)  Other adult smoker at home  No – ref  Yes OR = 2.18 (95% CI 0.92 – 5.14) |
| Peltzer [[59](#_ENREF_59)] |  | E | Neither parent/guardian smokes – ref  Both parents/guardians smoke OR 5.45 (95% CI 2.67 – 8.1)  Father/male guardian smokes OR 4.25 (95% CI 3.41 – 5.3)  Mother/female guardian smokes OR 6.62 (95% CI 4.09 – 10.71) |
| Rachiotis et al.[[46](#_ENREF_46)] |  | E | Neither parent smoked – ref  Both parents smoked OR 2.86 (95% CI 2.35 – 3.32)  Father only smoked OR 2.08 (95% CI 1.76 – 2.46)  Mother only smoked OR 2.34 (95% CI 1.87 – 2.94) |
| Raisamo et al.[[51](#_ENREF_51)] |  | E | 12-14 year olds:  Neither parent smokes – ref  Mother smokes OR 6.9 (95% CI 5.1 – 8.2)  Father smokes OR 5.8 (95% CI 5.1 – 6.7)  Both parents smoke OR 13.5 (95% CI 11.1 – 15.5)  16-18 year olds:  Neither parent smokes – ref  Mother smokes OR 3.2 (95% CI 2.1 – 3.5)  Father smokes OR 2.9 (95% CI 2.1 – 3.1)  Both parents smoke OR 5.6 (95% CI 5.1 – 6.1) |
| Ulbricht et al.[[43](#_ENREF_43)] |  | E | Two parent household – one smoker - ref  Two parent household – two smokers OR 2.77 (95% CI 1.9 – 4.05), p < 0.001  Single parent household – one smoker OR 2.74 (95% CI 1.59 – 4.71), p < 0.001 |
| Hughes et al.[[48](#_ENREF_48)] |  | G | Respondent or spouse a smoker – yes OR 2.65 (95% CI 1.29 – 5.43) |
| Rudatsikira et al.[[58](#_ENREF_58)] |  | G | Parents smoking  None – ref  Father only OR 3.65 (95% CI 3.1 – 4.3)  Mother only OR 6.54 (95% CI 3.48 – 12.32)  Both parents OR 5.85 (95% CI 3.83 – 8.92) |
| *Number of cigarettes smoked* | | | |
| Bakoula et al.[[44](#_ENREF_44)] | Cigarettes per day | B | Increase of 10 cig/day = 37% increase (95% CI 32-43) |
| Dell’Orco et al.[[53](#_ENREF_53)] | Cigarettes per day | B | None – ref  Only father (1-10) ratio 1.36 (95% CI 1.14-1.63)  Only mother (1-10) ratio 1.60 (95% CI 1.14-1.63)  Both (1-10) ratio 2.17 (95% CI 1.62-2.90)  Only father (> 10) ratio 1.99 (95% CI 1.77-2.24)  Only mother (>10) ratio 2.44 (95% CI 1.93-3.09)  Both (father (>10) and mother (1-10)) ratio 2.44 (95% CI 2.07-2.88)  Both (father (1-10) and mother (>10)) ratio 2.37 (95% CI 1 69-3.31)  Both (>10) ratio 2.97 (95% CI 2.49-3.53)  Unknown ratio 1.60 (95% CI 1.26-2 02) |
| Mantziou et al.[[45](#_ENREF_45)] | Paternal cigarettes per day  Spouse non-smoker  Maternal cigarettes per day | E, F | Paternal smoking in house in front of children  B coefficient = 0.12, OR = 1.13 (95% CI 1.08 – 1.19) p < 0.001  B coefficient = -0.82, OR = 0.44 (95% CI 0.24 – 0.8), p = 0.007  Maternal smoking in the house in front of children  B coefficient = 0.04, OR = 0.01 (95% CI 1.00 – 1.08), p = 0.019 |
| Johansson et al.[[50](#_ENREF_50)] | Cigarettes per day | E | OR 1.6 (95% CI 1.2 – 2.1), p < .01 |
| *Number of cigarettes smoked in the home* | | | |
| Mills et al.[[29](#_ENREF_29)] | Maternal cigarettes smoked in the home | A  D | †  Salivary cotinine, p <.05  Time-weighted average PM_2.5,_ p <.05  Maximum, p <.05  % time over 35 µg/m^3^, p <.05 |
| Scherer et al.[[42](#_ENREF_42)] | Cigarettes per day smoked in home | B | †  % explained variance (R^2^)  0 or missing, geometric mean = 10.6, p < 0.001  5 geometric mean = 10.3  6-10 geometric mean =29.4  11-20 geometric mean = 38.0  21 geometric mean =67.8 |
| Mannino et al.[[39](#_ENREF_39)] | Cigarettes smoked in home | C | †  Mean increase in log cotinine, ng/ml  Unknown 0.82 (95% CI 0.64 – 1.00)  1-9 – ref  10-19 0.86 (95% CI 0.62 – 1.10)  20-29 1.14 (95% CI 0.86 – 1.32)  30-39 1.33 (95% CI 0.87 – 1.79)  ≥40 1.55 (95% CI 1.25 – 1.85) |
| *Attitudes to smoking/SHS exposure* | | | |
| Mills et al.[[29](#_ENREF_29)] | Maternal attitudes | C  A | †  Agreement with statement ‘I would ask a smoker to smoke outside my house’:  Maximum particulate matter was significantly lower in homes of mothers who strongly agreed, compared to no strong opinion (p = .03), disagreed (p = .034) or strongly disagreed (p = 0.013)  Salivary cotinine significantly higher in children of mothers who strongly disagreed compared to mothers who agreed (p = .004)or strongly agreed (p = .008)  Other attitudinal questions were non-significant |
| Peltzer[[59](#_ENREF_59)] | Child attitudes | E | Do you think the smoke from other people’s cigarettes is harmful to you?  Definitely not - ref  Probably not OR 1.38 (95% CI 0.98 – 1.95)  Probably yes OR 1.96 (95% CI 1.39 – 2.75)  Definitely yes OR 2.01 (95% CI 1.57 – 2.6) |
| Raute et al.[[60](#_ENREF_60)] | Child attitudes | E | Awareness about harmfulness of exposure to SHS from other people  Yes – ref  No adjusted OR = 1.68 (95% CI 1.15 – 2.45) |
| Rise & Lund[[52](#_ENREF_52)] | Attitudes towards SHS (range of questions, lower scores reflecting more negative attitudes towards SHS exposure) | E | 1995: β = 0.19, p < 0.05  2001: β = 0.41, p < 0.0001 |
| Soliman et al.[[33](#_ENREF_33)] | SHS harmful\not harmful\unsure | E | SHS exposure harmful OR 0.27 (95% CI 0.23-0.32)  Don’t know if SHS exposure is harmful OR 0.66 (95% CI 0.54-0.81) |
| Liao et al.[[61](#_ENREF_61)] | Scale measuring parental perceptions, evaluations of the consequences and family reactions to smoking in the presence of children | E | † Disagreed with home smoking bans OR 2.16 (95% CI 1.18-3.94)  Parental smokers reaction to family’s anti-smoking responses scale (lower score showing more compliance with family’s antismoke responses) OR 0.94 (95% CI 0.91-0.96) |
| *Family characteristics, family size* | | | |
| Jarvis et al.[[30](#_ENREF_30)] | Number of children in household | A | β = 0.79 (95% CI 0.72 – 0.87) (higher exposure in smaller families) |
| Bolte & Fromme[[41](#_ENREF_41)] | Family size | E | 1 child – ref  2 children OR 0.68 (95% CI 0.61 – 0.76)  ≥ 3 children OR 0.66 (95% CI 0.59 – 0.75) |
| Longman & Passey[[56](#_ENREF_56)] | Household size | E | 1–2 people, ref  3–4 people, OR 1.20 (95% CI 1.06 to 1.36)  5+ people, OR 1.36 (95% CI 1.08 to 1.72) |
| Hawkins & Berkman[[35](#_ENREF_35)] | Number of children | F | Smoking mothers:  Infant with no siblings – ref  Infant with 1 sibling OR 1.25 (95% CI 1.10, 1.41)  Infant with 2+ siblings OR 1.59 (95% CI 1.40, 1.81) |
| Ulbricht et al.[[43](#_ENREF_43)] | Number of children | E | 1 child – ref  ≥ 3 children OR 1.76 (95% CI 1.09 – 2.82), p = 0.019 |
| *Marital status* | | | |
| Jarvis et al.[[30](#_ENREF_30)] | One parent household | A | No β = 2.97 (95% CI 2.32 – 3.81) |
| Chen et al.[[37](#_ENREF_37)] | Single/not single | B | Single OR = 1.67 (95% CI 1.03 – 2.71)  Not single – ref |
| Preston et al.[[64](#_ENREF_64)] | Mother living alone | B | Living alone parameter estimates = -0.22 (95% CI -0.41 - -0.02), p = 0.027  Living with partner – ref |
| Bolte & Fromme[[41](#_ENREF_41)] | Single parent family/other | E | Single parent OR 1.38 (95% CI 1.20–1.57) |
| Raisamo et al.[[51](#_ENREF_51)] | Intact family/other | E | 12-14 year olds  Non-intact family OR 1.9 (95% CI 1.1–2.2) |
| Singh et al.[[38](#_ENREF_38)] | Two parent biological/two parent step family/single mother/other family type | E | Two parent biological – ref  Two-parent step family OR 1.35 (95% CI 1.12 – 1.63)  Single mother OR 1.72 (95% CI 1.43 – 2.07)  Other family type OR 2.07 (95% CI 1.60 – 2.69) |
| Hawkins & Berkman[[35](#_ENREF_35)] | Married/not married | F | Smoking mothers:  Married – ref  Not married – OR 1.13 (95% CI 1.01-1.27) |
| *Home characteristics* | | | |
| *Crowding* | | | |
| Sims 2010[[5](#_ENREF_5)] | (>1.5 people per bedroom) | A | 4-15 year olds, Adjusted for age and year:  People per bedroom <1 ref  1-1.5 β 0.277 (95% CI 0.185-0.369)  > 1.5 β 0.555 (95% CI 0.452-0.658) |
| Jarvis et al.[[30](#_ENREF_30)] | Persons per room | A | Β = 1.29 (95% CI 1.16 – 1.44) (more crowded experience greater exposure) |
| Dell’Orco et al.[[53](#_ENREF_53)] | Inhabitant’s per room | B | Low (<1) – ref  Medium (1-2) 1.08 (95% CI 0.96 – 1.22)  High (>2) 1.38 (95% CI 1.14 – 1.67)  Unknown 1.29 (95% CI 0.93 – 1.77) |
| Scherer et al.[[42](#_ENREF_42)] | Bedroom sharing | B | †  % explained by variance (R2) = 0.7, p < 0.01  Bedroom sharing geometric mean = 30.7  No bedroom sharing geometric mean = 26.7 |
| *Size of home* | | | |
| Bakoula et al.[[44](#_ENREF_44)] | Floor surface area | B | Floor surface area increase of 20 m2 -9% decrease (95% CI -14- -5) |
| Scherer et al.[[42](#_ENREF_42)] | Size of flat | B | †  % explained by variance (R2) = 10.5, p < 0.001  60m^2^ geometric mean = 42.4  60-120m^2^ geometric mean = 32.0  >120m^2^ geometric mean = 31.1 |
| Mannino et al.[[39](#_ENREF_39)] | Number of rooms | C | Mean increase in log cotinine, ng/ml  ≤5 0.27 (95% CI 0.07 – 0.47)  ≥6 – ref |
| *Air conditioning* | | | |
| Abidin et al.[[57](#_ENREF_57)] | Air conditioning in living room, child’s sleeping area or none | A | None – ref  Living room standard β = -0.11, p = 0.002  Child’s sleeping area standard β = -0.08, p = 0.017* |
| *Outside space available* | | | |
| Bleakley et al.[[36](#_ENREF_36)] |  | E | Access to outside space, OR 0.24 (95% CI 0.06 – 0.98) |
| Ulbricht et al.[[43](#_ENREF_43)] |  | E | No access to outside space, OR 4.38 (95% CI 2.64 – 7.25), p < 0.001 |
| *Child characteristics* | | | |
| *Age* | | | |
| Mills et al.[[29](#_ENREF_29)] |  | A | Β = -0.276, p < 0.05 ( |
| Cook et al.[[32](#_ENREF_32)] |  | A | T Test for trend = 3.8, p = 0.003 (younger children have higher geometric mean cotinine) |
| Delpisheh et al.[[25](#_ENREF_25)] |  | A | < 7 years OR = 1.9 (95% CI 1.4 – 2.6) (children < 7 years of age have higher salivary cotinine) |
| Sims et al.[[5](#_ENREF_5)] |  | A | Β = -0.025 (95% CI -0.031—0.018) (younger children have higher salivary cotinine) |
| Mannino et al.[[39](#_ENREF_39)] |  | C | Age years, mean increase in log cotinine, (ng/ml) (younger children have higher serum cotinine)  4-6 – 0.53 (95% CI 0.37 – 0.69)  7-11 – 0.17 (95% CI 0.03 – 0.31)  12-16 – ref |
| Baheiraei et al.[[63](#_ENREF_63)] |  | B | Per month increase in age OR 1.19 (95% CI 1.04-1.36) (older infants higher urinary cotinine) |
| Bakoula et al.[[44](#_ENREF_44)] |  | B | Age -9% decrease per year increase in age (95% CI 95% CI -11- -8) (younger children have higher urinary cotinine) |
| Preston et al.[[64](#_ENREF_64)] |  | B | (younger children have higher urinary cotinine)  2-4 years – ref  5-8 years parameter estimates = -0.32 (95% CI -0.5 - -0.13), p < 0.001  9-12 years parameter estimates = -0.42 (95% CI -0.62 - -0.22), p < 0.001 |
| Mantziou et al.[[45](#_ENREF_45)] |  | E | Paternal smoking in the house in front of children:  B coefficient = -0.12, OR = 0.89 (95% CI 0.8 – 0.99), p = 0.026 (younger children more likely to be exposed) |
| Rachiotis et al.[[46](#_ENREF_46)] |  | E | Age years:  11-13 - ref  14 OR 1.02 (95% CI 0.87 – 1.19)  15 OR 1.43 (95% CI 1.2 – 1.72)  16-17 OR 1.29 (1.13 – 2.18) |
| Rudatsikira et al.[[58](#_ENREF_58)] |  | E | (older children more likely to be exposed)  11-12 – ref  13 OR 0.97 (95% CI 0.72 – 1.32)  14 OR 1.27 (95% CI 0.94 – 1.73)  15 OR 1.41 (95% CI 1.04 – 1.92)  16-17 OR 1.53 (95% CI 1.03 – 2.26) |
| Bleakley et al.[[36](#_ENREF_36)] |  | E | Child under 5 years OR 0.38 (95% CI 0.17 – 0.82) (younger children less likely to be exposed) |
| *Gender* | | | |
| Cook et al.[[32](#_ENREF_32)] |  | A | T test for trend = 2.5, p = 0.01 (girls have higher geometric mean cotinine levels) |
| Jarvis et al.[[30](#_ENREF_30)] |  | A | Β 1.28 (95% CI 1.11 – 1.47) (girls have higher cotinine levels) |
| Sims et al.[[5](#_ENREF_5)] |  | A | 4-12 years olds  Female – 7% increase (regression coefficient = 0.068, 95% CI 0.02 – 0.1) |
| Bakoula et al.[[44](#_ENREF_44)] |  | B | Male -13% decrease (95% CI -21- -3) |
| Rachiotis et al.[[46](#_ENREF_46)] |  | E | Female – ref  Male OR 0.72 (0.62 – 0.81) |
| *Nursery attendance* | | | |
| Ulbricht et al.[[43](#_ENREF_43)] |  | E | No attendance at nursery OR 1.81 (95% CI 1.21 – 2.70), p < 0.001 |

1. Salivary cotinine
2. Urinary cotinine
3. Serum cotinine
4. Airborne particulate matter
5. SHS exposure in the home (parental/respondent/child reported)
6. Smoking in the presence of children
7. SHS exposure all locations (not limited to home)
